# Supplementary material for: Genetic Variation between Dengue Virus Type 4 Strains Impacts Human Antibody Binding and Neutralization
Source: Cell Rep. 2018 Oct 30;25(5):1214–24. doi: 10.1016/j.celrep.2018.10.006 (PMC6226424; doi:10.1016/j.celrep.2018.10.006)
Supplement: Document S1. Figures S1–S7 and Tables S1–S3 [file mmc1.pdf]

Cell Reports, Volume *25*

Supplemental Information

Genetic Variation between Dengue Virus Type 4 Strains Impacts Human Antibody Binding and Neutralization

Emily N. Gallichotte, Thomas J. Baric, Usha Nivarthi, Matthew J. Delacruz, Rachel Graham, Douglas G. Widman, Boyd L. Yount, Anna P. Durbin, Stephen S. Whitehead, Aravinda M. de Silva, and Ralph S. Baric

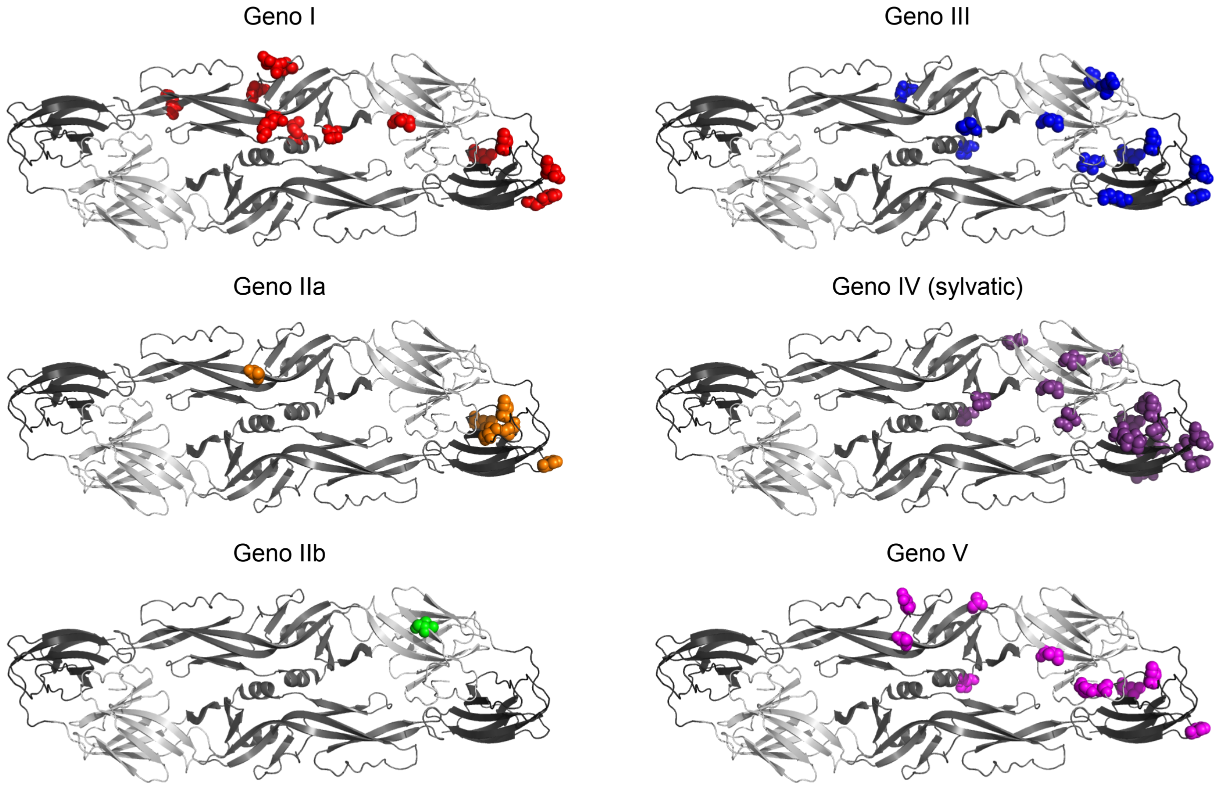

Supplemental Figure 1. Amino acid diversity of each DENV4 genotype virus. Related to Figure 2. Differences in envelope protein amino acid sequence of each genotype relative to WT (genotype IIb) were mapped on envelope protein dimer (PBD = 1OAN).

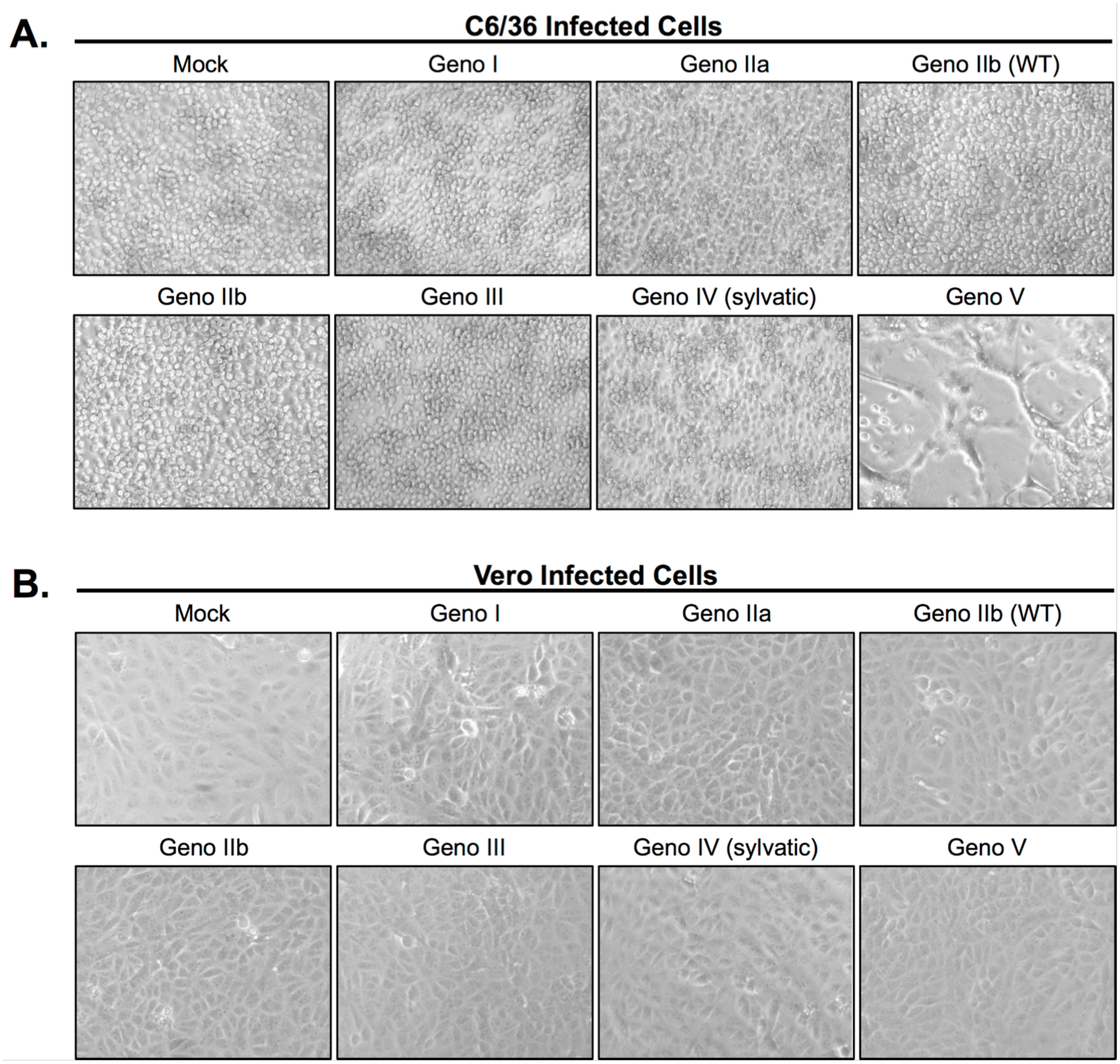

Supplemental Figure 2. Cytopathic effect of cells infected with DENV4 viruses. Related to Figure 3. A) C6/36 and B) Vero cells were infected at a multiplicity of infection (MOI) of 0.5 and imaged four days post-infection.

Virus	1	2	3	4	5	6	7	8	9	10	11	12	13	14	15	16	17	18	19	20	21	22	23	24	25	26	27	28	29	30	31	32	33	34	35	36	37	38	39	40	41	42	43	44	45	46
Geno I	F	H	L	S	T	R	D	G	E	P	L	M	I	V	A	K	H	E	R	G	R	P	L	L	F	K	T	T	E	G	I	N	K	C	T	L	I	A	M	D	L	G	E	M	C	E
Geno IIa*	F	H	L	S	T	R	D	G	E	P	L	M	I	V	A	K	H	E	R	G	R	P	L	L	F	K	T	T	E	G	I	N	K	C	T	L	I	A	M	D	L	G	E	M	C	E
Geno IIb (WT)	F	H	L	S	T	R	D	G	E	P	L	M	I	V	A	K	H	E	R	G	R	P	L	L	F	K	T	T	E	G	I	N	K	C	T	L	I	A	M	D	L	G	E	M	C	E
Geno IIb	F	H	L	S	T	R	D	G	E	P	L	M	I	V	A	K	H	E	R	G	R	P	L	L	F	K	T	T	E	G	I	N	K	C	T	L	I	A	M	D	L	G	E	M	C	E
Geno III*	F	H	L	S	T	R	D	G	E	P	L	M	I	V	A	K	H	E	R	G	R	P	L	L	F	K	T	T	E	G	I	N	K	C	T	L	I	A	M	D	L	G	E	M	C	E
Geno IV (sylvatic)	F	H	L	S	S	R	D	G	E	P	L	M	I	V	A	K	H	E	R	G	R	P	L	L	F	K	T	T	E	G	I	N	K	C	T	L	I	A	M	D	V	G	E	M	C	E
Geno V	F	H	L	S	T	R	D	G	E	P	L	M	I	V	A	K	H	E	R	G	R	P	L	L	F	K	T	T	E	G	I	N	K	C	T	L	I	A	M	D	L	G	E	M	C	E

Virus	47	48	49	50	51	52	53	54	55	56	57	58	59	60	61	62	63	64	65	66	67	68	69	70	71	72	73	74	75	76	77	78	79	80	81	82	83	84	85	86	87	88	89	90	91
Geno I	D	T	V	T	Y	K	C	P	L	L	V	N	T	E	P	E	D	I	D	C	W	C	N	L	T	S	A	W	V	M	Y	G	T	C	T	Q	S	G	E	R	R	R	E	K	R
Geno IIa*	D	T	V	T	Y	K	C	P	L	L	V	N	T	E	P	E	D	I	D	C	W	C	N	L	T	S	T	W	V	M	Y	G	T	C	T	Q	S	G	E	R	R	R	E	K	R
Geno IIb (WT)	D	T	V	T	Y	K	C	P	L	L	V	N	T	E	P	E	D	I	D	C	W	C	N	L	T	S	T	W	V	M	Y	G	T	C	T	Q	S	G	E	R	R	R	E	K	R
Geno IIb	D	T	V	T	Y	K	C	P	L	L	V	N	T	E	P	E	D	I	D	C	W	C	N	L	T	S	T	W	V	M	Y	G	T	C	T	Q	S	G	E	R	R	R	E	K	R
Geno III*	D	T	V	T	Y	K	C	P	L	L	V	N	T	E	P	E	D	I	D	C	W	C	N	L	T	S	T	W	V	M	Y	G	T	C	T	Q	N	G	E	R	R	R	E	K	R
Geno IV (sylvatic)	D	T	V	T	Y	K	C	P	L	L	V	N	T	E	P	E	D	I	D	C	W	C	N	S	T	S	T	W	V	T	Y	G	T	C	T	Q	S	G	E	R	R	R	E	K	R
Geno V	D	T	V	M	Y	K	C	P	L	L	V	N	T	E	P	E	D	I	D	C	W	C	N	L	T	S	T	W	V	M	Y	G	T	C	T	Q	S	G	E	R	R	R	E	K	R

Supplemental Figure 3. Sequence alignment of pr protein. Related to Figure 4. For the genotype IIa and III (*) envelope sequences used in our panel, paired pr sequences were not are available. Consensus pr sequences based on those available from other viruses within genotypes IIa and III in the phylogenetic tree are shown.

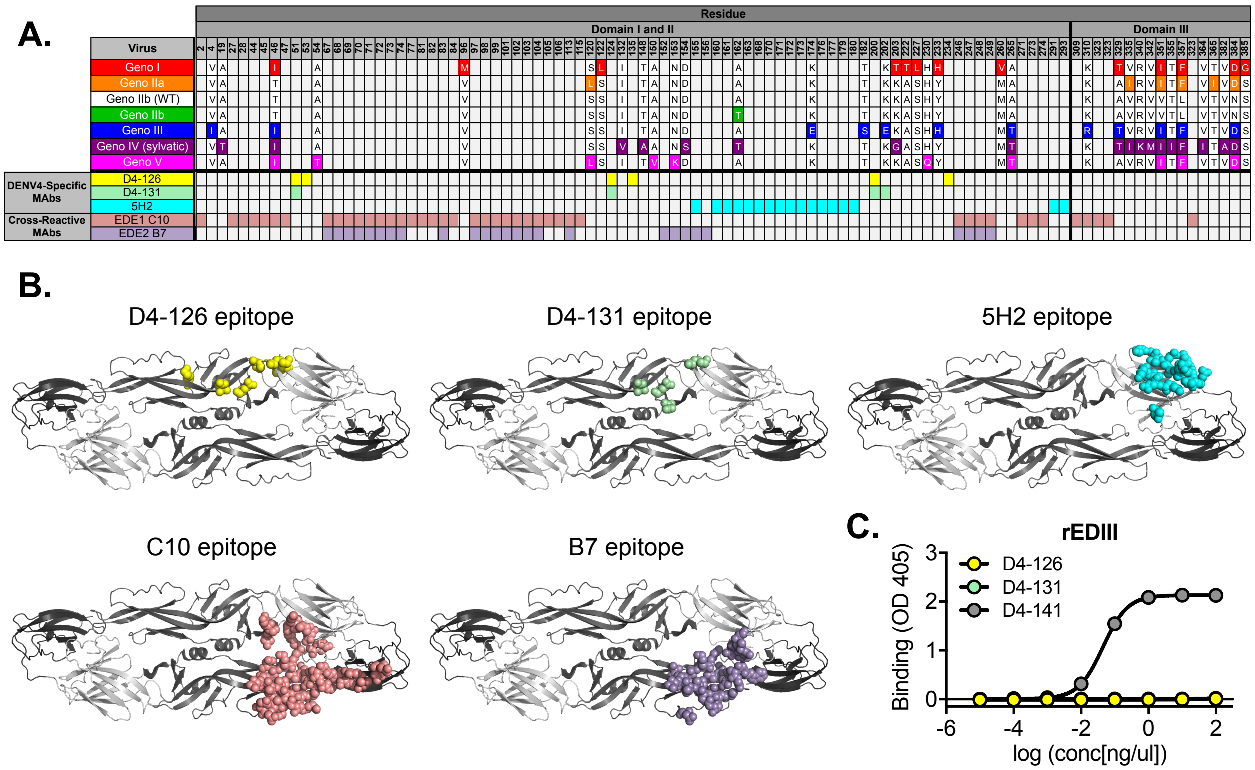

Supplemental Figure 4. Epitopes of DENV serotype-specific and cross-reactive antibodies. Related to Figures 5-6. A) Amino acid sequences of DENV4 genotype viruses with known monoclonal antibodies contact residues highlighted. B) Monoclonal antibody contact residues are mapped onto the envelope dimer (PBD = 1OAN). C) Monoclonal antibody D4-141 binds recombinant envelope domain III (rEDIII) protein.

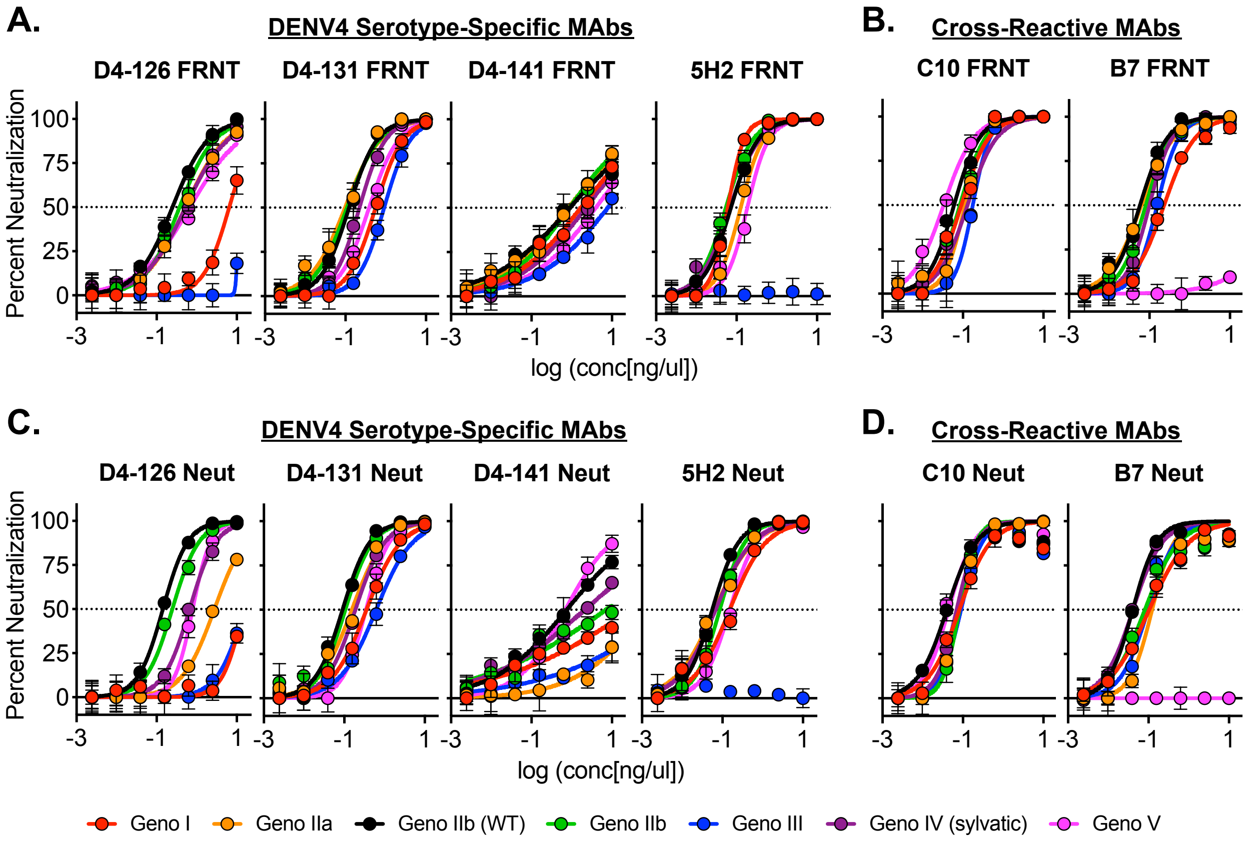

Supplemental Figure 5. Monoclonal antibody neutralization curves. Related to Figure 6. DENV4 serotype-specific antibodies D4-126, D4-131, D4-141 and 5H2 and DENV cross-reactive antibodies C10 and B7 were evaluated for their ability to neutralize DENV4 genotype viruses in a A) Vero cell focus reduction neutralization test (FRNT) and B) flow-cytometery-based neutralization assay (Neut) (mean ± SD of biological triplicates).

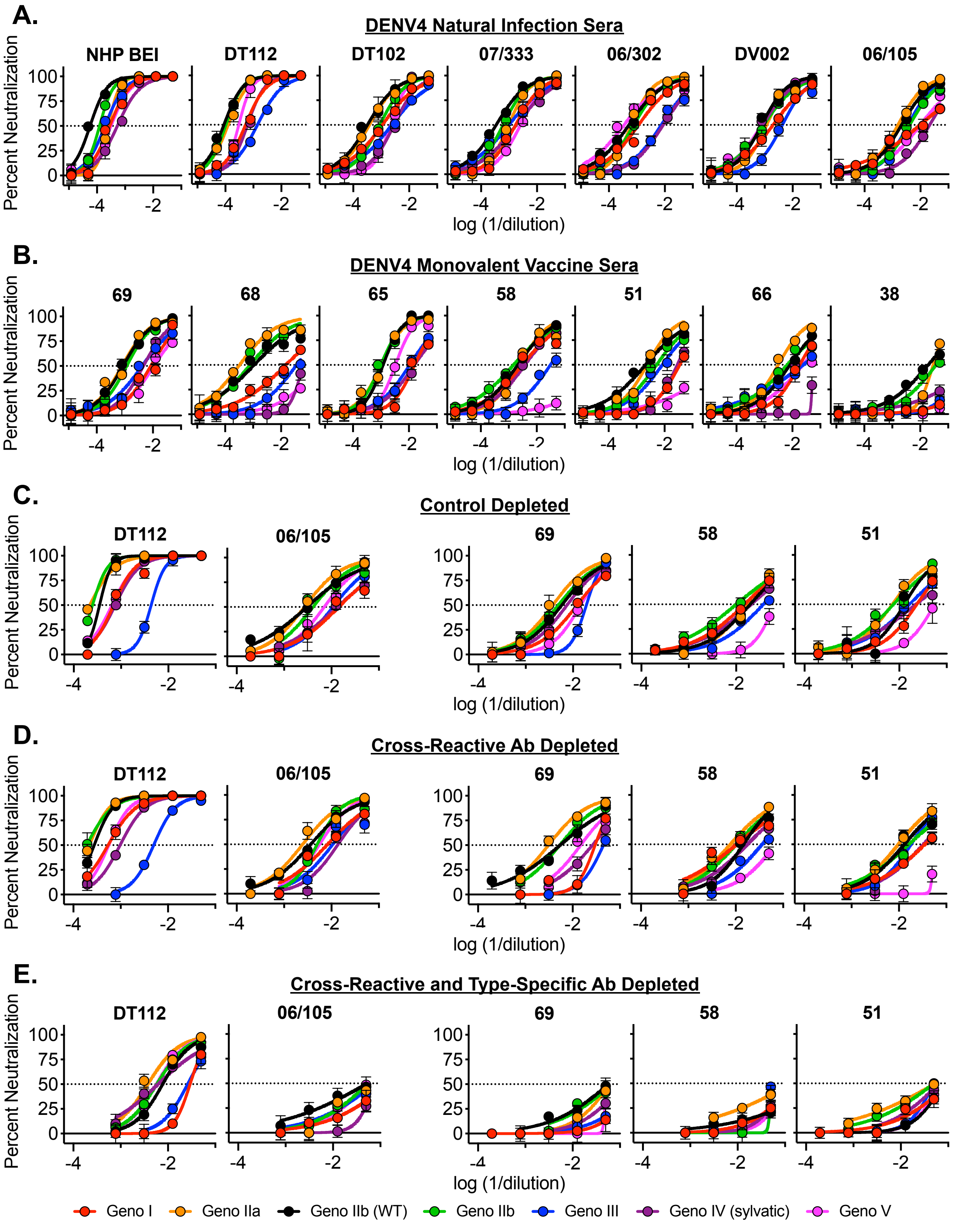

Supplemental Figure 6. DENV4 natural infection and monovalent vaccine immune sera neutralization curves. Related to Figure 7. Using Vero cell focus reduction neutralization test (FRNT), A) pooled polyclonal immune sera

from DENV4 infected non-human primates (NHP) or from naturally infected individuals, or from B) individuals who received the NIH DENV4 monovalent vaccine were evaluated for their ability to neutralize DENV4 genotype viruses. DENV4 natural infection and monovalent vaccine sera were C) control depleted with BSA, D) depleted of cross-reactive antibodies, or E) depleted of all DENV antibodies and evaluated for their ability to neutralize DENV4 genotype viruses (mean ± SD of biological duplicates).

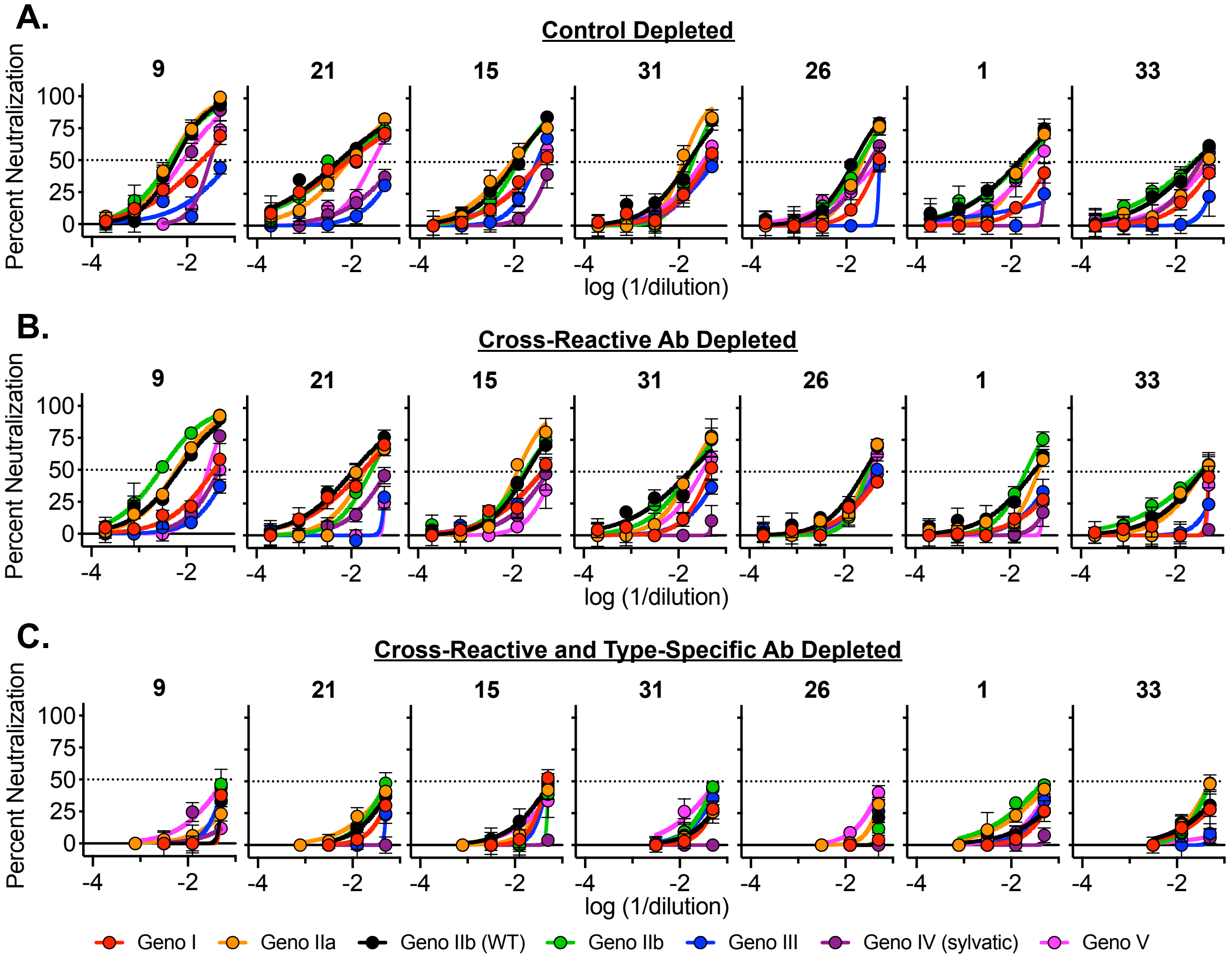

Supplemental Figure 7. DENV tetravalent vaccine immune sera neutralization curves. Related to Figure 7. Using Vero cell focus reduction neutralization test (FRNT), DENV tetravalent immune sera were A) control depleted with BSA, B) depleted of cross-reactive antibodies, or C) depleted of all DENV antibodies and evaluated for their ability to neutralize DENV4 genotype viruses (mean ± SD of biological duplicates).

Genotype	Location	Year	Accession Number
Genotype I	Cambodia	2010	KF543272.1
Genotype IIa	French Polynesia	2009	JN832541.1
Genotype IIb	Puerto Rico	1999	FJ882599.1
Genotype IIb (WT)	Sri Lanka	1992	KJ160504.1
Genotype III	Thailand	2001	AY618940.1
Genotype IV	Malaysia	1973	AF231724.1
Genotype V	India	1961	JF262783.1

Supplemental Table 1. Viruses in recombinant DENV4 variant panel. Related to Figures 1-2.

Name	Description	Region	Genotype
D4/13DX3-741_JX644006	Dengue virus 4 strain 13DX3-741 envelope protein gene, partial cds	Vietnam	I
D4/13DX3-773_JX644009	Dengue virus 4 strain 13DX3-773 envelope protein gene, partial cds	Vietnam	I
D4/13DX3-779_JX644008	Dengue virus 4 strain 13DX3-779 envelope protein gene, partial cds	Vietnam	I
D4/13DX4-413_JX644007	Dengue virus 4 strain 13DX4-413 envelope protein gene, partial cds	Vietnam	I
D4/13DX4-417_JX644010	Dengue virus 4 strain 13DX4-417 envelope protein gene, partial cds	Vietnam	I
D4/13DX4-446_JX644011	Dengue virus 4 strain 13DX4-446 envelope protein gene, partial cds	Vietnam	I
D4/13DX4-452_JX644012	Dengue virus 4 strain 13DX4-452 envelope protein gene, partial cds	Vietnam	I
D4/China/YN/15DGR284/2015_KX262925	Dengue virus 4 isolate DENV-4/China/YN/15DGR284 (2015) envelope protein gene, partial cds	China	I
D4/China/YN/15DGR32/2015_KX262923	Dengue virus 4 isolate DENV-4/China/YN/15DGR32 (2015) envelope protein gene, partial cds	China	I
D4/China/YN/15DGR34/2015_KX262920	Dengue virus 4 isolate DENV-4/China/YN/15DGR34 (2015) envelope protein gene, partial cds	China	I
D4/China/YN/15DGR35/2015_KX262921	Dengue virus 4 isolate DENV-4/China/YN/15DGR35 (2015) envelope protein gene, partial cds	China	I
D4/China/YN/15DGR394/2015_KX262926	Dengue virus 4 isolate DENV-4/China/YN/15DGR394 (2015) envelope protein gene, partial cds	China	I
D4/China/YN/15DGR50/2015_KX262924	Dengue virus 4 isolate DENV-4/China/YN/15DGR50 (2015) envelope protein gene, partial cds	China	I
D4/China/YN/15DGR9/2015_KX262922	Dengue virus 4 isolate DENV-4/China/YN/15DGR9 (2015) envelope protein gene, partial cds	China	I
D4/H781363_JQ513345	Dengue virus 4 strain H781363, complete genome	Brazil	I
D4/Hu/Cambodia/NRTqs/2013_AB873105	Dengue virus 4 gene for envelope protein, partial cds, strain: D4/Hu/Cambodia/NRTqs/2013	Cambodia	I
D4/SG(EHI)D4/02990Y14_KX224312	Dengue virus 4 isolate SG(EHI)D4/02990Y14, complete genome	Singapore	I
D4/U0811386_KF543272	Dengue virus 4 isolate U0811386 polyprotein gene, partial cds	Cambodia	I
D4/CO/BID-V3406/2001_GQ868579	Dengue virus 4 isolate DENV-4/CO/BID-V3406/2001, complete genome	Colombia	IIa
D4/CO/BID-V3410/2004_GQ868583	Dengue virus 4 isolate DENV-4/CO/BID-V3410/2004, complete genome	Colombia	IIa
D4/CO/BID-V3411/2004_GQ868584	Dengue virus 4 isolate DENV-4/CO/BID-V3411/2004, complete genome	Colombia	IIa
D4/CO/BID-V3412/2005_GQ868585	Dengue virus 4 isolate DENV-4/CO/BID-V3412/2005, complete genome	Colombia	IIa
D4/H772846_JQ513330	Dengue virus 4 strain H772846, complete genome	Brazil	IIa
D4/H772852_JQ513331	Dengue virus 4 strain H772852, complete genome	Brazil	IIa
D4/H772854_JN559741	Dengue virus 4 strain H772854, complete genome	Brazil	IIa
D4/H773583_JQ513332	Dengue virus 4 strain H773583, complete genome	Brazil	IIa
D4/H774846_JQ513333	Dengue virus 4 strain H774846, complete genome	Brazil	IIa
D4/H779228_JQ513338	Dengue virus 4 strain H779228, complete genome	Brazil	IIa
D4/H779652_JQ513339	Dengue virus 4 strain H779652, complete genome	Brazil	IIa
D4/H780090_JQ513340	Dengue virus 4 strain H780090, complete genome	Brazil	IIa
D4/H780120_JQ513341	Dengue virus 4 strain H780120, complete genome	Brazil	IIa
D4/H780556_JQ513342	Dengue virus 4 strain H780556, complete genome	Brazil	IIa
D4/H780563_JQ513343	Dengue virus 4 strain H780563, complete genome	Brazil	IIa
D4/H780571_JQ513344	Dengue virus 4 strain H780571, complete genome	Brazil	IIa
D4/MT/BR12_TVP17898/2012_KJ579243	Dengue virus 4 strain DENV-4/MT/BR12_TVP17898/2012 isolate serum_12, complete genome	Brazil	IIa
D4/MT/BR2_TVP17888/2012_KJ579240	Dengue virus 4 strain DENV-4/MT/BR2_TVP17888/2012 isolate serum_2, complete genome	Brazil	IIa
D4/MT/BR20_TVP17906/2012_KJ579244	Dengue virus 4 strain DENV-4/MT/BR20_TVP17906/2012 isolate serum_20, complete genome	Brazil	IIa
D4/MT/BR23_TVP17909/2012_KJ579245	Dengue virus 4 strain DENV-4/MT/BR23_TVP17909/2012 isolate serum_23, complete genome	Brazil	IIa
D4/MT/BR24_TVP17910/2012_KJ579246	Dengue virus 4 strain DENV-4/MT/BR24_TVP17910/2012 isolate serum_24, complete genome	Brazil	IIa
D4/MT/BR27_TVP17913/2012_KJ579247	Dengue virus 4 strain DENV-4/MT/BR27_TVP17913/2012 isolate serum_27, complete genome	Brazil	IIa
D4/MT/BR28_TVP17914/2012_KJ579248	Dengue virus 4 strain DENV-4/MT/BR28_TVP17914/2012 isolate serum_28, complete genome	Brazil	IIa
D4/MT/BR33_TVP17919/2012_KJ596658	Dengue virus 4 strain DENV-4/MT/BR33_TVP17919/2012 isolate serum_33, complete genome	Brazil	IIa
D4/MT/BR35_TVP17921/2012_KJ596659	Dengue virus 4 strain DENV-4/MT/BR35_TVP17921/2012 isolate serum_35, complete genome	Brazil	IIa
D4/MT/BR40_TVP17926/2012_KJ596660	Dengue virus 4 strain DENV-4/MT/BR40_TVP17926/2012 isolate serum_40, complete genome	Brazil	IIa
D4/MT/BR44_TVP17930/2012_KJ596661	Dengue virus 4 strain DENV-4/MT/BR44_TVP17930/2012 isolate serum_44, complete genome	Brazil	IIa
D4/MT/BR47_TVP17933/2012_KJ596662	Dengue virus 4 strain DENV-4/MT/BR47_TVP17933/2012 isolate serum_47, complete genome	Brazil	IIa
D4/MT/BR48_TVP17934/2012_KJ596663	Dengue virus 4 strain DENV-4/MT/BR48_TVP17934/2012 isolate serum_48, complete genome	Brazil	IIa
D4/MT/BR50_TVP18148/2012_KJ596664	Dengue virus 4 strain DENV-4/MT/BR50_TVP18148/2012 isolate serum_50, complete genome	Brazil	IIa
D4/MT/BR52_TVP17938/2012_KJ596665	Dengue virus 4 strain DENV-4/MT/BR52_TVP17938/2012 isolate serum_52, complete genome	Brazil	IIa
D4/MT/BR53_TVP17939/2012_KJ596666	Dengue virus 4 strain DENV-4/MT/BR53_TVP17939/2012 isolate serum_53, complete genome	Brazil	IIa
D4/MT/BR55_TVP17941/2012_KJ596667	Dengue virus 4 strain DENV-4/MT/BR55_TVP17941/2012 isolate serum_55, complete genome	Brazil	IIa
D4/MT/BR60_TVP17946/2012_KJ596668	Dengue virus 4 strain DENV-4/MT/BR60_TVP17946/2012 isolate serum_60, complete genome	Brazil	IIa
D4/MT/BR73_TVP17951/2012_KJ596669	Dengue virus 4 strain DENV-4/MT/BR73_TVP17951/2012 isolate serum_73, complete genome	Brazil	IIa
D4/MT/BR76_TVP17953/2012_KJ596670	Dengue virus 4 strain DENV-4/MT/BR76_TVP17953/2012 isolate serum_76, complete genome	Brazil	IIa
D4/MT/BR8_TVP17894/2012_KJ579241	Dengue virus 4 strain DENV-4/MT/BR8_TVP17894/2012 isolate serum_8, complete genome	Brazil	IIa
D4/MT/BR84_TVP17961/2012_KJ596671	Dengue virus 4 strain DENV-4/MT/BR84_TVP17961/2012 isolate serum_84, complete genome	Brazil	IIa
D4/MT/BR9_TVP17895/2012_KJ579242	Dengue virus 4 strain DENV-4/MT/BR9_TVP17895/2012 isolate serum_9, complete genome	Brazil	IIa
D4/MT/BR91_TVP17968/2012_KJ596672	Dengue virus 4 strain DENV-4/MT/BR91_TVP17968/2012 isolate serum_91, complete genome	Brazil	IIa
D4/MT/BR92_TVP17969/2012_KJ596673	Dengue virus 4 strain DENV-4/MT/BR92_TVP17969/2012 isolate serum_92, complete genome	Brazil	IIa
D4/MT/BR94_TVP17971/2012_KJ596674	Dengue virus 4 strain DENV-4/MT/BR94_TVP17971/2012 isolate serum_94, complete genome	Brazil	IIa
D4/PF09/220709-54_JN832541	Dengue virus 4 isolate PF09/220709-54 envelope protein (E) gene, partial cds	French Polynesia	IIa
D4/SG(EHI)D4/20293Y13_KX224301	Dengue virus 4 isolate SG(EHI)D4/20293Y13 envelope protein gene, partial cds	Singapore	IIa
D4/SG(EHI)D4/21661Y13_KX224302	Dengue virus 4 isolate SG(EHI)D4/21661Y13 envelope protein gene, partial cds	Singapore	IIa
D4/SG(EHI)D4/25974Y13_KX224304	Dengue virus 4 isolate SG(EHI)D4/25974Y13 envelope protein gene, partial cds	Singapore	IIa
D4/SG(EHI)D4/30313Y13_KX224311	Dengue virus 4 isolate SG(EHI)D4/30313Y13 envelope protein gene, partial cds	Singapore	IIa
D4/SG/06K2270DK1/2005_GQ398256	Dengue virus 4 strain DENV-4/SG/06K2270DK1/2005, complete genome	Singapore	IIa
D4/VE/BID-V1153/2007_GQ868642	Dengue virus 4 isolate DENV-4/VE/BID-V1153/2007, complete genome	Venezuela	IIa
D4/VE/BID-V1154/2007_GQ868643	Dengue virus 4 isolate DENV-4/VE/BID-V1154/2007, complete genome	Venezuela	IIa
D4/VE/BID-V1155/2007_GQ868644	Dengue virus 4 isolate DENV-4/VE/BID-V1155/2007, complete genome	Venezuela	IIa
D4/VE/BID-V1156/2007_GQ868645	Dengue virus 4 isolate DENV-4/VE/BID-V1156/2007, complete genome	Venezuela	IIa
D4/VE/BID-V1157/2007_EU854299	Dengue virus 4 isolate DENV-4/VE/BID-V1157/2007, complete genome	Venezuela	IIa
D4/VE/BID-V1158/2007_FJ182016	Dengue virus 4 isolate DENV-4/VE/BID-V1158/2007, complete genome	Venezuela	IIa
D4/VE/BID-V1159/2007_EU854300	Dengue virus 4 isolate DENV-4/VE/BID-V1159/2007, complete genome	Venezuela	IIa
D4/VE/BID-V1160/2007_FJ182017	Dengue virus 4 isolate DENV-4/VE/BID-V1160/2007, complete genome	Venezuela	IIa
D4/VE/BID-V1161/2007_EU854301	Dengue virus 4 isolate DENV-4/VE/BID-V1161/2007, complete genome	Venezuela	IIa
D4/VE/BID-V2163/1998_FJ639736	Dengue virus 4 isolate DENV-4/VE/BID-V2163/1998, complete genome	Venezuela	IIa
D4/VE/BID-V2164/1998_FJ639737	Dengue virus 4 isolate DENV-4/VE/BID-V2164/1998, complete genome	Venezuela	IIa
D4/VE/BID-V2165/1998_FJ639738	Dengue virus 4 isolate DENV-4/VE/BID-V2165/1998, complete genome	Venezuela	IIa
D4/VE/BID-V2166/1998_FJ639739	Dengue virus 4 isolate DENV-4/VE/BID-V2166/1998, complete genome	Venezuela	IIa
D4/VE/BID-V2167/1998_JN819409	Dengue virus 4 isolate DENV-4/VE/BID-V2167/1998, complete genome	Venezuela	IIa
D4/VE/BID-V2170/1999_FJ639742	Dengue virus 4 isolate DENV-4/VE/BID-V2170/1999, complete genome	Venezuela	IIa
D4/VE/BID-V2172/1999_FJ639744	Dengue virus 4 isolate DENV-4/VE/BID-V2172/1999, complete genome	Venezuela	IIa
D4/VE/BID-V2173/1999_FJ639745	Dengue virus 4 isolate DENV-4/VE/BID-V2173/1999, complete genome	Venezuela	IIa
D4/VE/BID-V2176/2000_FJ850095	Dengue virus 4 isolate DENV-4/VE/BID-V2176/2000, complete genome	Venezuela	IIa
D4/VE/BID-V2177/2000_FJ639748	Dengue virus 4 isolate DENV-4/VE/BID-V2177/2000, complete genome	Venezuela	IIa
D4/VE/BID-V2194/2001_FJ639764	Dengue virus 4 isolate DENV-4/VE/BID-V2194/2001, complete genome	Venezuela	IIa
D4/VE/BID-V2206/2001_FJ639773	Dengue virus 4 isolate DENV-4/VE/BID-V2206/2001, complete genome	Venezuela	IIa
D4/VE/BID-V2489/2007_FJ882580	Dengue virus 4 isolate DENV-4/VE/BID-V2489/2007, complete genome	Venezuela	IIa

D4/VE/BID-V2490/2007_FJ882581	Dengue virus 4 isolate DENV-4/VE/BID-V2490/2007, complete genome	Venezuela	IIa
D4/VE/BID-V2491/2007_FJ882582	Dengue virus 4 isolate DENV-4/VE/BID-V2491/2007, complete genome	Venezuela	IIa
D4/VE/BID-V2492/2007_FJ882583	Dengue virus 4 isolate DENV-4/VE/BID-V2492/2007, complete genome	Venezuela	IIa
D4/VE/BID-V2493/200_FJ882584	Dengue virus 4 isolate DENV-4/VE/BID-V2493/2007, complete genome	Venezuela	IIa
D4/VE/BID-V2494/2007_FJ882585	Dengue virus 4 isolate DENV-4/VE/BID-V2494/2007, complete genome	Venezuela	IIa
D4/VE/BID-V2495/2007_FJ882586	Dengue virus 4 isolate DENV-4/VE/BID-V2495/2007, complete genome	Venezuela	IIa
D4/VE/BID-V2496/2007_FJ882587	Dengue virus 4 isolate DENV-4/VE/BID-V2496/2007, complete genome	Venezuela	IIa
D4/VE/BID-V2497/2007_FJ882588	Dengue virus 4 isolate DENV-4/VE/BID-V2497/2007, complete genome	Venezuela	IIa
D4/VE/BID-V2498/2007_FJ882589	Dengue virus 4 isolate DENV-4/VE/BID-V2498/2007, complete genome	Venezuela	IIa
D4/VE/BID-V2499/2007_FJ882590	Dengue virus 4 isolate DENV-4/VE/BID-V2499/2007, complete genome	Venezuela	IIa
D4/VE/BID-V2500/2007_FJ882591	Dengue virus 4 isolate DENV-4/VE/BID-V2500/2007, complete genome	Venezuela	IIa
D4/VE/BID-V2501/2008_FJ882592	Dengue virus 4 isolate DENV-4/VE/BID-V2501/2008, complete genome	Venezuela	IIa
D4/VE/BID-V2607/2006_JN819406	Dengue virus 4 isolate DENV-4/VE/BID-V2607/2006, complete genome	Venezuela	IIa
D4/VE/BID-V2610/2007_GQ199876	Dengue virus 4 isolate DENV-4/VE/BID-V2610/2007, complete genome	Venezuela	IIa
D4/ARC-1-13_KF809760	Dengue virus 4 strain ARC-1-13 envelope protein gene, partial cds	Puerto Rico	IIb
D4/ARC-33-13_KF809761	Dengue virus 4 strain ARC-33-13 envelope protein gene, partial cds	Puerto Rico	IIb
D4/ARC-55-12_KF809754	Dengue virus 4 strain ARC-55-12 envelope protein gene, partial cds	Puerto Rico	IIb
D4/ARC-65-12_KF809755	Dengue virus 4 strain ARC-65-12 envelope protein gene, partial cds	Puerto Rico	IIb
D4/ARC-65-13_KF809762	Dengue virus 4 strain ARC-65-13 envelope protein gene, partial cds	Puerto Rico	IIb
D4/ARC-69-12_KF809756	Dengue virus 4 strain ARC-69-12 envelope protein gene, partial cds	Puerto Rico	IIb
D4/ARC-75-12_KF809757	Dengue virus 4 strain ARC-75-12 envelope protein gene, partial cds	Puerto Rico	IIb
D4/ARC-78-12_KF809758	Dengue virus 4 strain ARC-78-12 envelope protein gene, partial cds	Puerto Rico	IIb
D4/ARC-80-12_KF809759	Dengue virus 4 strain ARC-80-12 envelope protein gene, partial cds	Puerto Rico	IIb
D4/CO/BID-V1600/1997_FJ024476	Dengue virus 4 isolate DENV-4/CO/BID-V1600/1997, complete genome	Colombia	IIb
D4/CO/BID-V3407/2001_GQ868580	Dengue virus 4 isolate DENV-4/CO/BID-V3407/2001, complete genome	Colombia	IIb
D4/CO/BID-V3408/2001_GQ868581	Dengue virus 4 isolate DENV-4/CO/BID-V3408/2001, complete genome	Colombia	IIb
D4/CO/BID-V3409/2001_GQ868582	Dengue virus 4 isolate DENV-4/CO/BID-V3409/2001, complete genome	Colombia	IIb
D4/H402276_JN559740	Dengue virus 4 strain H402276, complete genome	Brazil	IIb
D4/H775222_JQ513334	Dengue virus 4 strain H775222, complete genome	Brazil	IIb
D4/H778494_JQ513335	Dengue virus 4 strain H778494, complete genome	Brazil	IIb
D4/H778504_JQ513336	Dengue virus 4 strain H778504, complete genome	Brazil	IIb
D4/H778887_JQ513337	Dengue virus 4 strain H778887, complete genome	Brazil	IIb
D4/rDENV4_KJ160504	Dengue virus 4 isolate rDENV4, complete genome	Sri Lanka	IIb
D4/US/BID-V1082/1998_FJ024424	Dengue virus 4 isolate DENV-4/US/BID-V1082/1998, complete genome	USA	IIb
D4/US/BID-V1083/1986_EU854295	Dengue virus 4 isolate DENV-4/US/BID-V1083/1986, complete genome	USA	IIb
D4/US/BID-V1093/1998_EU854296	Dengue virus 4 isolate DENV-4/US/BID-V1093/1998, complete genome	USA	IIb
D4/US/BID-V1094/1998_EU854297	Dengue virus 4 isolate DENV-4/US/BID-V1094/1998, complete genome	USA	IIb
D4/US/BID-V2429/1994_GQ199878	Dengue virus 4 isolate DENV-4/US/BID-V2429/1994, complete genome	USA	IIb
D4/US/BID-V2430/1994_GQ199879	Dengue virus 4 isolate DENV-4/US/BID-V2430/1994, complete genome	USA	IIb
D4/US/BID-V2431/1995_GQ199880	Dengue virus 4 isolate DENV-4/US/BID-V2431/1995, complete genome	USA	IIb
D4/US/BID-V2432/1995_GQ252675	Dengue virus 4 isolate DENV-4/US/BID-V2432/1995, complete genome	USA	IIb
D4/US/BID-V2433/1995_FJ810417	Dengue virus 4 isolate DENV-4/US/BID-V2433/1995, complete genome	USA	IIb
D4/US/BID-V2434/1995_FJ850057	Dengue virus 4 isolate DENV-4/US/BID-V2434/1995, complete genome	USA	IIb
D4/US/BID-V2435/1996_GQ199881	Dengue virus 4 isolate DENV-4/US/BID-V2435/1996, complete genome	USA	IIb
D4/US/BID-V2436/1996_GQ199882	Dengue virus 4 isolate DENV-4/US/BID-V2436/1996, complete genome	USA	IIb
D4/US/BID-V2437/1996_GQ199883	Dengue virus 4 isolate DENV-4/US/BID-V2437/1996, complete genome	USA	IIb
D4/US/BID-V2438/1996_GQ199884	Dengue virus 4 isolate DENV-4/US/BID-V2438/1996, complete genome	USA	IIb
D4/US/BID-V2439/1996_GQ199885	Dengue virus 4 isolate DENV-4/US/BID-V2439/1996, complete genome	USA	IIb
D4/US/BID-V2440/1996_FJ850058	Dengue virus 4 isolate DENV-4/US/BID-V2440/1996, complete genome	USA	IIb
D4/US/BID-V2441/1998_FJ882595	Dengue virus 4 isolate DENV-4/US/BID-V2441/1998, complete genome	USA	IIb
D4/US/BID-V2442/1998_FJ882596	Dengue virus 4 isolate DENV-4/US/BID-V2442/1998, complete genome	USA	IIb
D4/US/BID-V2443/1998_FJ850059	Dengue virus 4 isolate DENV-4/US/BID-V2443/1998, complete genome	USA	IIb
D4/US/BID-V2444/1998_FJ882597	Dengue virus 4 isolate DENV-4/US/BID-V2444/1998, complete genome	USA	IIb
D4/US/BID-V2445/1998_FJ882598	Dengue virus 4 isolate DENV-4/US/BID-V2445/1998, complete genome	USA	IIb
D4/US/BID-V2446/1999_FJ882599	Dengue virus 4 isolate DENV-4/US/BID-V2446/1999, complete genome	USA	IIb
D4/US/BID-V2447/1999_FJ882600	Dengue virus 4 isolate DENV-4/US/BID-V2447/1999, complete genome	USA	IIb
D4/US/BID-V2448/1999_FJ882601	Dengue virus 4 isolate DENV-4/US/BID-V2448/1999, complete genome	USA	IIb
D4/US/BID-V860/1994_FJ226067	Dengue virus 4 isolate DENV-4/US/BID-V860/1994, complete genome	USA	IIb
D4/0017/1997_AY618978	Dengue virus type 4 strain ThD4_0017_97 envelope protein (E) gene, partial cds	Thailand	III
D4/0164/1999_AY618986	Dengue virus type 4 strain ThD4_0164_99 envelope protein (E) gene, partial cds	Thailand	III
D4/0439/2001_AY618940	Dengue virus type 4 strain ThD4_0439_01 envelope protein (E) gene, partial cds	Thailand	III
D4/0476/1997_AY618979	Dengue virus type 4 strain ThD4_0476_97 envelope protein (E) gene, partial cds	Thailand	III
D4/1270/1998_AY618981	Dengue virus type 4 strain ThD4_1270_98 envelope protein (E) gene, partial cds	Thailand	III
D4/P73-1120_AF231724	Dengue virus type 4 isolate P73-1120 envelope protein (E) gene, partial cds	Malaysia	IV (sylvatic)
D4/P75-215_AF231725	Dengue virus type 4 isolate P75-215 envelope protein (E) gene, partial cds	Malaysia	IV (sylvatic)
D4/P75-514_AF231723	Dengue virus type 4 isolate P75-514 envelope protein (E) gene, partial cds	Malaysia	IV (sylvatic)
D4/Hu/Thailand/1963_DVU18440	Dengue virus type 4 Thailand 1963 polyprotein gene, partial cds	Thailand	V
D4/IND/0952326/2009_JQ922560	Dengue virus 4 isolate DENV-4/IND/0952326/2009, complete genome	India	V
D4/IND/624000/1962_JQ922558	Dengue virus 4 isolate DENV-4/IND/624000/1962, complete genome	India	V
D4/IND/793679/1979_JQ922559	Dengue virus 4 isolate DENV-4/IND/793679/1979, complete genome	India	V
D4/INDIA-G11337_JF262783	Dengue virus 4 isolate INDIA G11337, complete genome	India	V
D4/KBPV-VR-31_KP406806	Dengue virus 4 isolate DENV-4/KBPV-VR-31, complete genome	South Korea	V
D4/NIV-611319_JQ686059	Dengue virus 4 strain NIV_611319 envelope protein gene, partial cds	India	V
D4/NIV-62231_JQ686066	Dengue virus 4 strain NIV_62231 envelope protein gene, partial cds	India	V
D4/NIV-62235_JQ686065	Dengue virus 4 strain NIV_62235 envelope protein gene, partial cds	India	V
D4/NIV-624000_JQ686064	Dengue virus 4 strain NIV_624000 envelope protein gene, partial cds	India	V
D4/NIV-631302_JQ686057	Dengue virus 4 strain NIV_631302 envelope protein gene, partial cds	India	V
D4/NIV-64431_JQ686060	Dengue virus 4 strain NIV_64431 envelope protein gene, partial cds	India	V
D4/NIV-654021_JQ686062	Dengue virus 4 strain NIV_654021 envelope protein gene, partial cds	India	V
D4/NIV-654129-2_JQ686061	Dengue virus 4 strain NIV_654129-2 envelope protein gene, partial cds	India	V
D4/NIV-793679_JQ686058	Dengue virus 4 strain NIV_793679 envelope protein gene, partial cds	India	V
D4/PH/BID-V3361/1956_GQ868594	Dengue virus 4 isolate DENV-4/PH/BID-V3361/1956, complete genome	Philippines	V

Supplemental Table 2. DENV4 virus sequences in phylogenetic analyses. Related to Figure 1. All sequences used to generate phylogenetic tree in Figure 1 are listed by sequence name, description, location of collection and genotype.

Name	Asn-67 Gly Site	Asn-153 Gly Site	Asn-153 Seq.
D4/Hu/Thailand/1963_DVU18440	yes	**no**	**NDI**
D4/IND/0952326/2009_JQ922560	yes	yes	NDT
D4/IND/624000/1962_JQ922558	yes	**no**	**NDI**
D4/IND/793679/1979_JQ922559	yes	**no**	**NDI**
D4/INDIA-G11337_JF262783	yes	**no**	**KDT**
D4/KBPV-VR-31_KP406806	yes	**no**	**NDI**
D4/NIV-611319_JQ686059	yes	**no**	**NDI**
D4/NIV-62231_JQ686066	yes	**no**	**NDI**
D4/NIV-62235_JQ686065	yes	**no**	**KDT**
D4/NIV-624000_JQ686064	yes	**no**	**NDI**
D4/NIV-631302_JQ686057	yes	**no**	**NDI**
D4/NIV-64431_JQ686060	yes	**no**	**KDT**
D4/NIV-654021_JQ686062	yes	yes	NDT
D4/NIV-654129-2_JQ686061	yes	**no**	**NDI**
D4/NIV-793679_JQ686058	yes	**no**	**NDI**
D4/PH/BID-V3361/1956_GQ868594	yes	**no**	**NDI**
		**87.5% missing gly site**	

Supplemental Table 3. Glycosylation site sequences of all genotype V sequences. Related to Figure 4. 16 genotype V sequences used to generate phylogenetic tree in Figure 1 were evaluated to determine if glycosylation sites were present at positions 67 and 153. Fourteen of 16 sequences are missing glycosylation motif (N-X-T/S) at position 153.
