# Supplementary material for: Genetic Variation between Dengue Virus Type 4 Strains Impacts Human Antibody Binding and Neutralization
Source: Cell Rep. 2018 Oct 30;25(5):1214–24. doi: 10.1016/j.celrep.2018.10.006 (PMC6226424; doi:10.1016/j.celrep.2018.10.006)
Supplement: Document S2. Article plus Supplemental Information [file mmc2.pdf]

Cell Reports

Article

Genetic Variation between Dengue Virus Type 4 Strains Impacts Human Antibody Binding and Neutralization

Graphical Abstract

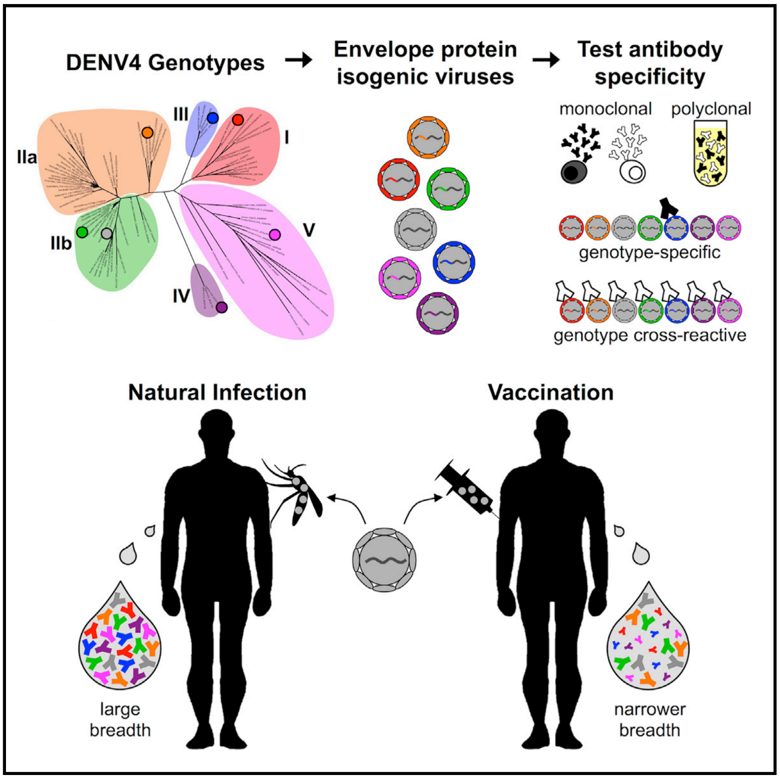

Highlights

- There is amino acid variability within the envelope protein across DENV4 genotypes
- DENV4 viruses differ in maturation, glycosylation, and ability to infect cells
- Monoclonal antibodies differentially bind and neutralize DENV4 genotype viruses
- Infection and vaccination elicit antibodies, which neutralize DENV4s differently

Authors

Emily N. Gallichotte, Thomas J. Baric, Usha Nivarthi, ..., Stephen S. Whitehead, Aravinda M. de Silva, Ralph S. Baric

Correspondence

egallich@email.unc.edu (E.N.G.),
rbaric@email.unc.edu (R.S.B.)

In Brief

Gallichotte et al. show that subtle genetic variation within the envelope protein across DENV4 genotype viruses can have disproportionally large impacts on many aspects of virus biology. Additionally, genotype viruses are differentially bound and neutralized by DENV antibodies, suggesting that DENV4 immunity may not be equally protective against all DENV4 viruses.

Gallichotte et al., 2018, Cell Reports *25*, 1214–1224
October 30, 2018 © 2018 The Author(s).
https://doi.org/10.1016/j.celrep.2018.10.006

OPEN ACCESS
CellPress

Cell Reports
Article

Genetic Variation between Dengue Virus Type 4 Strains Impacts Human Antibody Binding and Neutralization

Emily N. Gallichotte,[1,5,*] Thomas J. Baric,[2] Usha Nivarthi,[1] Matthew J. Delacruz,[1] Rachel Graham,[2] Douglas G. Widman,[2] Boyd L. Yount,[2] Anna P. Durbin,[3] Stephen S. Whitehead,[4] Aravinda M. de Silva,[1] and Ralph S. Baric[1,2,*]
[1]Department of Microbiology and Immunology, University of North Carolina at Chapel Hill School of Medicine, Chapel Hill, NC, USA
[2]Department of Epidemiology, University of North Carolina at Chapel Hill School of Public Health, Chapel Hill, NC, USA
[3]Johns Hopkins Bloomberg School of Public Health, Baltimore, MD, USA
[4]Laboratory of Viral Diseases, National Institute of Allergy and Infectious Diseases, National Institutes of Health, Bethesda, MD, USA
[5]Lead Contact
*Correspondence: egallich@email.unc.edu (E.N.G.), rbaric@email.unc.edu (R.S.B.)
https://doi.org/10.1016/j.celrep.2018.10.006

SUMMARY

There are four distinct DENV serotypes, and within DENV4, there are five distinct genotypes. The impact of genotypic diversity is not known, nor is it clear whether infection with one DENV4 genotype results in protective immunity against the other genotypes. To measure the impact of DENV4 genetic diversity, we generated an isogenic panel of viruses containing the envelope protein from the different genotypes. We characterized many properties of these viruses and find that a small number of amino acids changes within the envelope have disproportionate impacts on virus biology. Additionally, we observe large differences in the ability of DENV4 antibodies, immune sera, and vaccine sera to neutralize the panel, suggesting that DENV4 immunity might not be equally protective against all DENV4s. Our results support the monitoring of changing or emerging DENV genotypes and their role in escaping pre-existing neutralizing antibodies in people who have been vaccinated or exposed to natural DENV4 infections.

INTRODUCTION

Dengue virus (DENV) is a single-stranded positive sense RNA virus. It is estimated that over one-third of the world's population is at risk for DENV infection, resulting in almost 400 million infections annually (Bhatt et al., 2013). Infection with DENV can result in a range of symptoms, from subclinical or mild disease, to severe DENV hemorrhagic disease and shock syndrome (Halstead, 2015; Katzelnick et al., 2016). There are four genetically and antigenically distinct DENV serotypes (DENV1–DENV4), which co-circulate around the world (Weaver and Vasilakis, 2009; Calisher et al., 1989; Holmes and Twiddy, 2003). Infection with one serotype is thought to provide long-term protection against subsequent infection with the homologous serotype; however, individuals are at risk for infection with the remaining three serotypes (Coloma and Harris, 2015). However, there are rare instances of reinfection with the homologous serotype (Forshey et al., 2016; Waggoner et al., 2016), suggesting that homotypic immunity may fail to prevent infection under some conditions (Katzelnick et al., 2015).

The four DENV serotypes share approximately 80% homology at an amino acid level across the entire coding region of the genome (Fleith et al., 2016). The envelope glycoprotein is roughly 70% conserved across DENV1–DENV4, containing fully conserved regions with no variation (e.g., fusion loop), and other regions containing highly divergent sequences (Rey et al., 2018). The molecular and evolutionary drivers of variation between and within serotypes remains uncertain (Bennett et al., 2010; Holmes and Twiddy, 2003). As determined using phylogenetic analyses, within each serotype, there are multiple genetically distinct genotypes, which are more closely related to each other than they are to the other serotypes (Weaver and Vasilakis, 2009).

DENV4 was first reported in the Philippines and Thailand in 1953, has since spread worldwide, and currently co-circulates with DENV1–DENV3 (Messina et al., 2014). Within DENV4, there are five distinct genotypes (I, II, III, IV, and V) with genotype II being further divided into IIa and IIb (Figure 1) (Chen and Han, 2016). Genotypes I and II currently circulate in human populations throughout the world (Cao-Lormeau et al., 2011; Dash et al., 2011; Fares et al., 2015; Klungthong et al., 2004). Conversely, genotype III, IV, and V infections are relatively rare. Genotype III has been detected sporadically in Asia between 1997 and 2015, and genotype V was primarily detected in India in the 1960s, but has been detected as recently as 2009 (Klungthong et al., 2004; Zhao et al., 2010; Shihada et al., 2017). Genotype IV is sylvatic, with only three known sequences (Durbin et al., 2013; Rossi et al., 2012), and has not yet been shown to spillover into humans, although rare cases of transient spillover have been documented for DENV1–DENV3 (Teoh et al., 2010; Vasilakis et al., 2008b).

In this manuscript, we used reverse genetics to generate a panel of recombinant DENV4 viruses that contain an isogenic backbone and differ only by the genotype sequence of the E protein. We used this panel of viruses to evaluate biological and virological properties associated with the E protein including its impact on neutralization using a well-characterized panel of human monoclonal antibodies, convalescent DENV4 sera, and

This is an open access article under the CC BY license (http://creativecommons.org/licenses/by/4.0/).

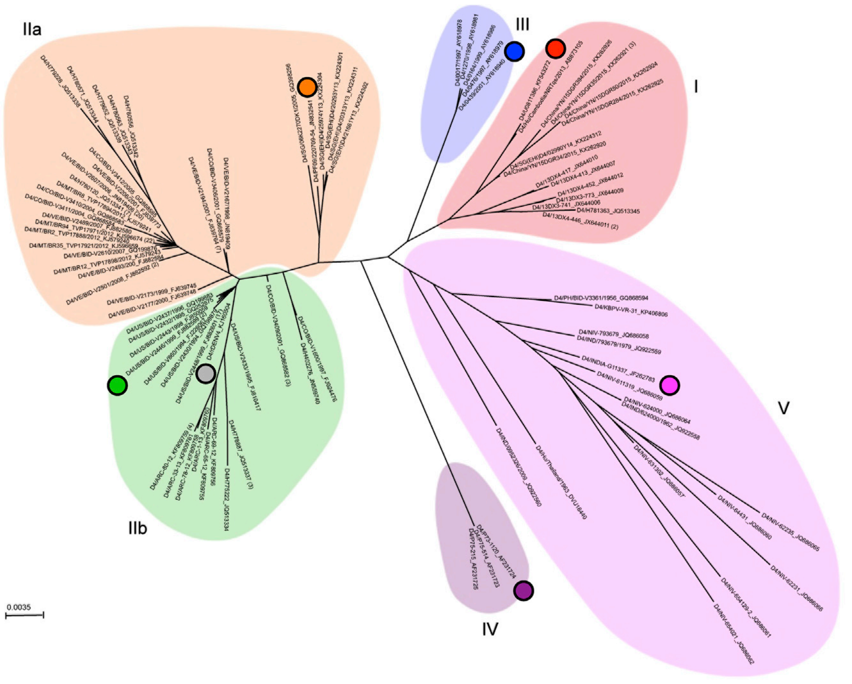

Figure 1. Phylogenetic Relationship of DENV4 Genotypes

DENV4 envelope protein sequences were aligned using neighbor-joining method with 100 replicates based on the multiple sequence alignment. Numbers in parentheses following virus species names indicate the number of sequences represented at that tree position.

vaccine sera from human volunteers. Our data reveal clear and significant antigenic differences among the DENV4 genotypes, which is critical for understanding immunity after natural DENV infection and evaluating vaccine responses.

RESULTS

Design of DENV4 Isogenic Envelope Panel

Phylogenetic analyses of DENV4 identifies six groups designated as genotypes I, IIa, IIb, III, IV (sylvatic), and V (Figure 1). As different isolates and genotypes of DENVs demonstrate variable growth rates and foci morphology in cell culture, hampering comparative studies of E protein variation, we used reverse genetics to construct a panel of recombinant DENV4 viruses. Using our previously described DENV4 molecular clone (genotype IIb) (Gallichotte et al., 2015), we replaced the wild-type (WT) envelope sequence with that from each of the other genotypes (Table S1; Figure 2A). All other structural and non-structural proteins were derived from WT DENV4, resulting in an isogenic panel of viruses that only differ in the E gene sequence (Figure 2A). Sequence analyses across the DENV4 genotype viruses reveal significant amino acid variation in EDIII as well as residues adjoining the hinge region between EDI and EDII (Figures 2B, 2C, and S1). When looking at the representative strain for each genotype, some amino acids differ in only one virus (e.g., position 132), whereas at other sites (e.g., position 351) the residues are variable across multiple genotypes (Figures 2B and 2C).

DENV4 Viruses Differ in Growth Kinetics and Foci Morphology

To recover recombinant viruses, full-length cDNAs were assembled as previously described (Gallichotte et al., 2015; 2017). Viruses were isolated by electroporating full-length infectious viral RNA into C6/36 cells, and passaging cell-culture supernatant once to produce infectious stocks. When C6/36 insect cells were infected in a multi-step viral growth curve (MOI of 0.01), all viruses replicated with similar kinetics and achieved similar peak titers of about 10^7 ffu/mL after 4 days, with genotype IIa having slightly lower titers at earlier time points (Figure 3A). Growth curves performed at a higher MOI in C6/36 cells had similar growth kinetics across the panel, although viruses reached peak titers by day 3 post-infection (Figure 3A). The recombinant DENVs displayed more heterogeneous growth kinetics on Vero cells following both low and high MOI infections (Figure 3B). Genotype IIb viruses replicated most efficiently, and genotype V was significantly attenuated in growth, with peak titers 3 logs lower than that of the other genotypes (Figure 3B). While genotype V was highly attenuated in Vero cells, it was the only virus to cause complete syncytia in C6/36 cells (Figure S2). Although speculative, it is possible that this demonstrates a virus adaptation for increased growth and spread in insect cells and insects. Syncytia formation has been seen with other strains of DENV (Pierro et al., 2006), but we did not observe syncytia with any of the other DENV4 genotype viruses.

In addition to virus growth, we also compared viral foci morphology (Figures 3C–3F). C6/36 foci were similar across the entire panel (Figures 3C and 3D). Slightly more variation in foci morphology and size was noted in Vero cells across the panel, with genotype V producing the smallest foci; however, all strains produced foci that were clearly defined and visible (Figures 3E and 3F). The attenuated foci size of genotype V was consistent with reduced replication in Vero cell growth curves (Figure 3B).

DENV4 E Genotype Viruses Do Not Differ in Thermostability

The large differences in viral replication of the DENV4 panel in mammalian and insect cells (Figure 3) may be a result of different temperature sensitivities, as studies have implicated envelope sequences and virion stability (Lim et al., 2017). A thermostability assay revealed that the DENV4 variants are similarly stable, with little loss of infectivity after incubation at 28°C and 37°C; however, all viruses lost ~1 log of infectivity after incubation at 40°C (Figure 4A). These results demonstrate that something other

A

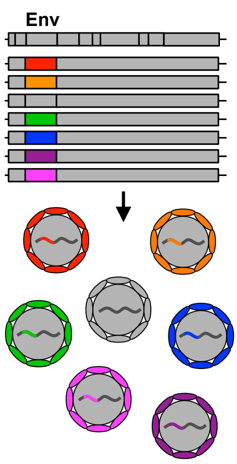

B

Virus	Residue																																			
	Domain I and II																							Domain III												
	4	19	46	54	96	120	122	132	148	150	153	154	162	174	182	202	203	222	227	230	233	260	265	310	329	335	340	342	351	355	357	364	365	382	384	385
Geno I	V	A	I	A	M	S	L	I	T	A	N	D	A	K	T	K	T	T	L	H	H	V	A	K	T	V	R	V	I	T	F	V	T	V	D	G
Geno IIa	V	A	T	A	V	L	S	I	T	A	N	D	A	K	T	K	K	A	S	H	Y	M	A	K	A	I	R	V	I	T	F	V	I	V	D	S
Geno IIb (WT)	V	A	T	A	V	S	S	I	T	A	N	D	A	K	T	K	K	A	S	H	Y	M	A	K	A	V	R	V	V	T	L	V	T	V	N	S
Geno IIb	V	A	T	A	V	S	S	I	T	A	N	D	T	K	T	K	K	A	S	H	Y	M	A	K	A	V	R	V	V	T	L	V	T	V	N	S
Geno III	I	A	I	A	V	S	S	I	T	A	N	D	A	E	S	E	K	A	S	H	H	M	T	R	T	V	R	V	I	T	F	V	T	V	D	S
Geno IV (sylvatic)	V	T	I	A	V	S	S	V	A	A	N	S	T	K	T	K	G	A	S	H	Y	M	T	K	T	I	K	M	I	I	F	I	T	A	D	S
Geno V	V	A	I	T	V	L	S	I	T	V	K	D	A	K	T	K	K	A	S	Q	Y	M	T	K	A	V	R	V	I	T	F	V	T	V	D	S

C

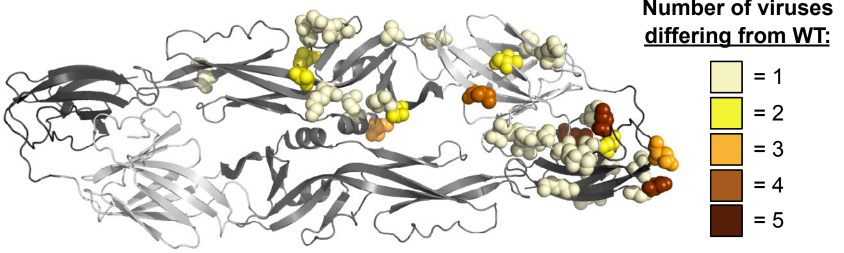

Figure 2. Design and Diversity of a Panel of DENV4 Genotypic Variants

(A) Sequences encoding the envelope protein from each genotype were placed into a genotype IIb (WT) infectious clone, resulting in recombinant viruses that are entirely WT except for envelope protein, and sequence encoding envelope protein.

(B) Envelope protein amino acids positions that differ from WT (genotype IIb).

(C) Envelope protein amino acids that differ from WT are mapped on dimer based on number of genotypes differing at that position (PDB: 1OAN).

than thermostability contributes to differences in the ability of the viruses to infect and replicate in mammalian and insect cells.

DENV4 E Variants Differ in Maturation Status, Enhanceability, and Glycosylation Pattern

As DENV maturation state may be heterogeneous *in vitro*, the recombinant panel allowed us to evaluate the role of E protein sequence on maturation status in an isogenic DENV4 backbone. During infection, DENV is assembled within the endoplasmic reticulum as immature virions containing pre-membrane (prM) proteins, which prevent fusion during viral egress. As DENV transits through the trans-Golgi network, pH change triggers cleavage of prM by the host protease furin. As the virus leaves the cell, cleaved pr dissociates, leaving fully mature viral particles. In cell culture, furin cleavage and pr dissociation are inefficient processes and highly cell type dependent, leading to heterogonous population of differentially mature viruses, containing different amounts of uncleaved pr peptide (Pierson and Diamond, 2012). As maturation status can influence infectivity and antibody neutralization (Mukherjee et al., 2014), we compared the maturation status across the DENV4 panel using immunoblotting and ELISAs (Figures 4B and 4C).

Immunoblotting revealed that the levels of pr protein varied across the panel, with genotype I and III being the least mature (most pr present) and genotypes IV and V being the most mature (very little pr protein detected) (Figure 4B). To corroborate these findings using a different assay, ELISA binding assays were performed, by capturing DENV4 viruses with cross-reactive monoclonal antibodies (mAbs) 4G2 (anti-E) and 2H2 (anti-pr), then probing with a pr-specific antibody (1E16) (Smith et al., 2015). These studies also demonstrated that there are differing levels of pr protein across the panel (Figure 4C). Consistent with immunoblotting, genotype III was highly immature, whereas genotype V was the most mature. As the DENV4 variant panel contains differing amounts of pr protein (Figures 4B and 4C), we sought to determine whether the viruses could be enhanced by a non-neutralizing, pr-specific mAb (Smith et al., 2015). An antibody-dependent enhancement (ADE) assay revealed that despite differing levels of pr present on viruses, all viruses are similarly enhanced, although the concentration of antibody needed to achieve peak enhancement and the level of enhancement do vary across the viruses (Figure 4D).

The furin cleavage site (located in prM protein) was not altered across the panel, suggesting that the E protein sequence can impact virus maturation (Pierson and Diamond, 2012). At a neutral pH of the released virus, the pr protein sits over the fusion-loop and is predicted to interact with seven amino acids in EDII (Figures 4E and 4F); however, at low pH, during processing of the virion, pr can make additional contacts across the envelope dimer. Under either condition, none of these amino acids were altered in the panel, suggesting that other residues may function to stabilize pr. Interestingly, there is little variability within the paired pr protein sequences of the variant panel (Figure S3).

Immunoblotting also revealed that the envelope protein of genotype V is slightly smaller than that of the other genotypes (Figure 4B). The DENV envelope protein contains two glycosylation sites, one in EDII (Asn-67) and one in EDI (Asn-153) (Figure 4E). Analysis of the glycosylation site sequences across the panel revealed that a single amino acid change at residue 153 in genotype V disrupts the N-X-T/S glycosylation motif (Figure 4G), resulting in a smaller molecular weight envelope protein (Figure 4B). When looking at all genotype V viruses used in our phylogenetic tree (Figure 1; Table S2), 87.5% have amino acid variability that disrupts the Asn-153 glycosylation site (Table S3), suggesting this disrupted motif is not unique

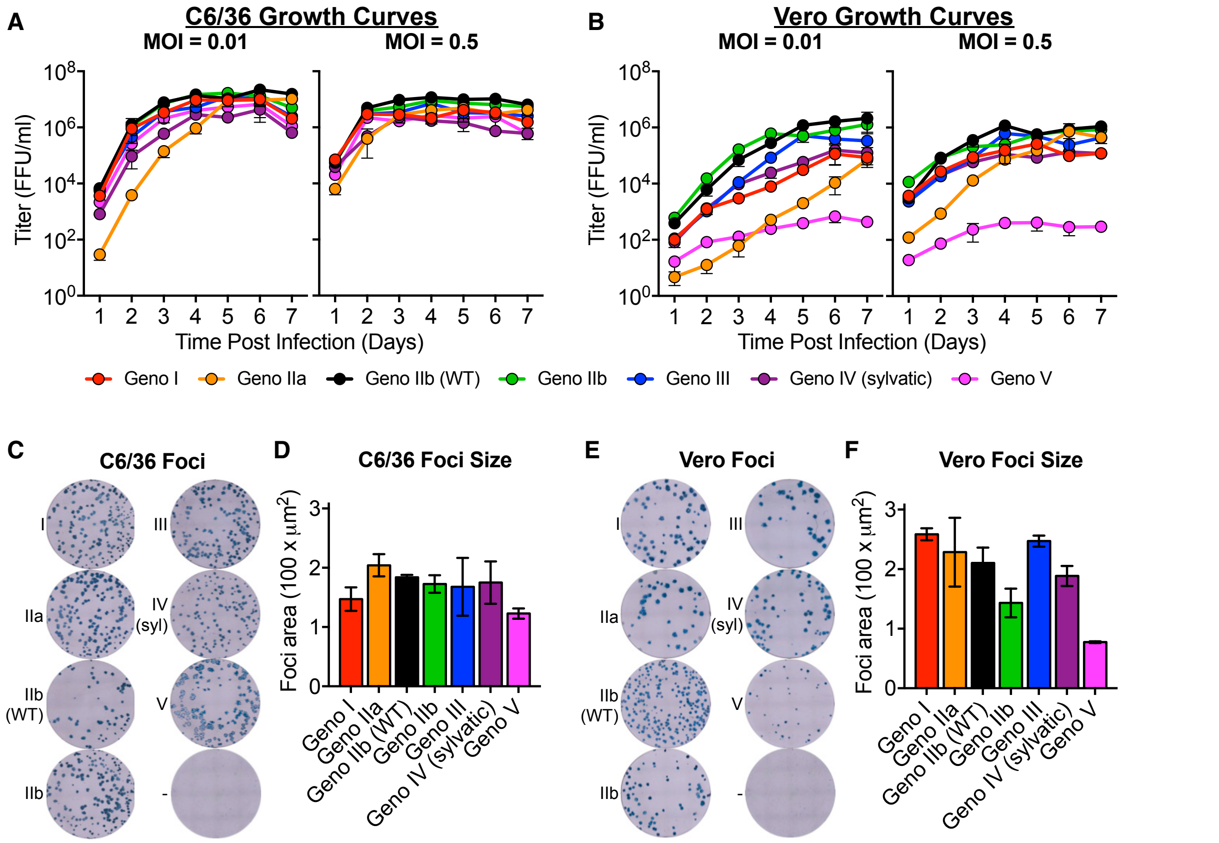

Figure 3. DENV4 Genotype Viruses Differ in Growth and Foci Morphology

(A and B) Viruses were analyzed for their ability to replicate in (A) C6/36 and (B) Vero cells at multiplicities of infection (MOI) of either 0.01 or 0.5 (mean ± SD of biological triplicates).

(C–F) Viral foci were immunostained on both (C) C6/36 and (E) Vero cells, and average foci area for (D) C6/36 and (F) Vero cells were calculated using CTL ImmunoSpot analyzer software (mean ± SD of biological triplicates).

to the genotype V strain selected within our panel, but appears to be conserved across most genotype V viruses. DENV envelope glycosylation can be important for binding to host cell receptors, determining infectivity in different hosts, and binding and neutralization by antibodies (Pokidysheva et al., 2006; Mondotte et al., 2007; Rouvinski et al., 2015). Therefore, genotype V's conserved lack of the second glycosylation site might impact the virus's ability to efficiently infect and be transmitted between vertebrate and invertebrate hosts, and be the result of adaptation to a different cellular or host tropism (Bryant et al., 2007; Lee et al., 2010). Additionally, the lack of this glycosylation site might contribute to the genotype V C6/36 syncytia phenotype (Figure S2). Genetic alteration of glycosylation and pr protein sequences, and generation of fully mature and fully immature virus preparations would allow one to determine the role of glycosylation and maturation status on many aspects of virus biology. Glycosylation status and large differences in viral maturation state have previously been shown to impact antibody binding and neutralization (Mukherjee et al., 2014); therefore, the binding and neutralization differences we see within this panel might be partially attributable to the variation in glycosylation and maturation.

Binding of Serotype-Specific and Cross-Reactive mAbs to DENV4 E Genotype Variants

We next measured the binding of a panel of well-characterized DENV4 serotype-specific and DENV cross-reactive mAbs to our DENV4 viruses by ELISA (Figure 5). DENV4 serotype-specific antibodies D4-126 and D4-131 recognize partially overlapping epitopes that have not been fully defined in the EDI/II hinge region (Figures S4A and S4B) (Nivarthi et al., 2017). All DENV4 genotypes bound similarly to D4-126 and D4-131 (Figure 5A). mAb D4-141, which recognizes an EDIII epitope (Figure S4C), also bound all viruses similarly, despite large amount of variation in EDIII across the panel (Figure 5A). The non-human primate mAb 5H2, which binds to a well-defined epitope on EDI, displayed highly variable binding across the panel (Figure 5A). Two amino acids (162 and 174) predicted to be 5H2 contact residues were variable across the DENV4 panel (Figure S4A) (Cockburn et al., 2012). Genotype III did not bind 5H2 and contains an amino acid polymorphism at position 174, suggesting that this position is essential for 5H2 binding.

The cross-reactive human mAbs C10 and B7 recognize quaternary envelope dimer epitopes (EDEs) that span across the fusion loop of one E monomer into EDIII or EDI of the neighboring

OPEN ACCESS
CellPress

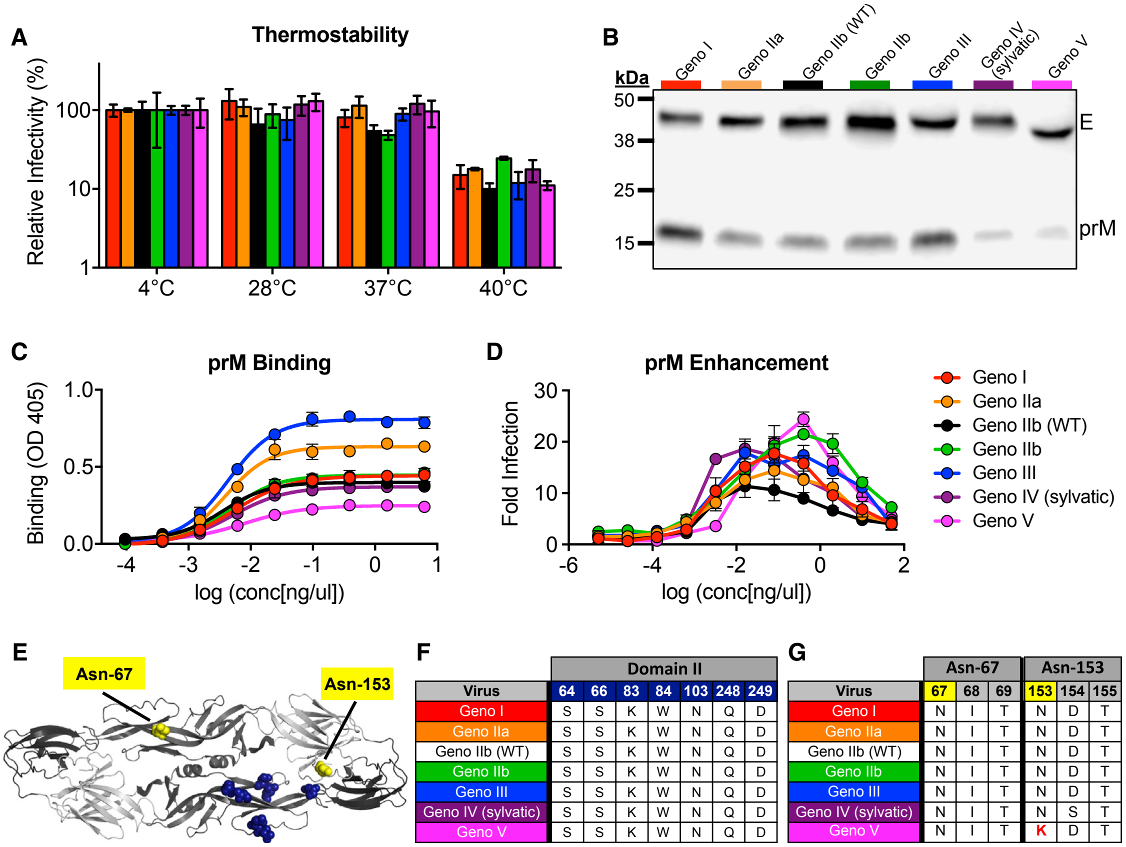

Virus	Domain II 64	66	83	84	103	248	249
Geno I	S	S	K	W	N	Q	D
Geno IIa	S	S	K	W	N	Q	D
Geno IIb (WT)	S	S	K	W	N	Q	D
Geno IIb	S	S	K	W	N	Q	D
Geno III	S	S	K	W	N	Q	D
Geno IV (sylvatic)	S	S	K	W	N	Q	D
Geno V	S	S	K	W	N	Q	D

Virus	Asn-67 67	68	69	Asn-153 153	154	155
Geno I	N	I	T	N	D	T
Geno IIa	N	I	T	N	D	T
Geno IIb (WT)	N	I	T	N	D	T
Geno IIb	N	I	T	N	D	T
Geno III	N	I	T	N	D	T
Geno IV (sylvatic)	N	I	T	N	S	T
Geno V	N	I	T	K	D	T

Figure 4. DENV4 Genotype Viruses Differ in Thermostability, Maturation, and Glycosylation Status

(A) DENV4 viruses were evaluated for their thermostability at 28°C, 37°C, and 40°C (mean ± SD of biological triplicates). Relative infectivity is calculated as ($ffu/mL_{test\ temperature}$ / $ffu/mL_{4°C}$) × 100.

(B) Viruses were immunoblotted for envelope (E) and precursor (prM) protein present in viral stocks (representative image).

(C) Binding assay using pr-specific mAb 1E16 to detect amount of pr protein present in virus stocks (mean ± SD of biological triplicates).

(D) Antibody-dependent enhancement (ADE) assay was performed with DENV4 virus panel and anti-pr mAb 1E16 in U937 cells (mean ± SD of biological duplicates).

(E) DENV envelope dimer showing location of putative pr interacting residues (navy) and glycosylation sites (yellow) (PDB: 1OAN).

(F) Envelope and putative pr interacting sites (via side-chain interactions) are listed for each genotype.

(G) Amino acid sequences of glycosylation sites 67 and 153 for each genotype. Genotype V lysine at amino acid position 153 (highlighted in red) disrupts glycosylation motif (N-X-T/S).

monomer (Figures S4A and S4B) (Rouvinski et al., 2015). These mAbs bind all four DENV serotypes, reflecting the highly conserved nature of the epitope across the DENV E protein. Consequently, it was not surprising that C10 and B7 bound all genotypes within the DENV4 panel with similar efficiencies (Figure 5B), as the differences between genotypes are smaller than those between serotypes. Binding of B7, however, is dependent on the presence of a glycan at position 153 in EDI (Rouvinski et al., 2015). The DENV4 genotype V virus in this panel, which lacked this glycosylation site (Figure 4E), failed to bind to the B7 antibody (Figure 5B).

Neutralization of DENV4 E Genotype Variants by Serotype-Specific and Cross-Reactive mAbs

Next, we evaluated the ability of DENV4 serotype-specific and cross-reactive mAbs to neutralize the DENV4 panel in a Vero cell focus reduction neutralization test (FRNT), and a flow cytometry-based neutralization assay with U937 cells expressing DC-SIGN, a known DENV attachment factor (Figures 6A, 6B, and S5). We observed a 1- to 2-log difference in antibody neutralization titers of the DENV4 serotype-specific antibodies against the DENV4 panel. Importantly, some mAbs were not able to neutralize select genotypes even at the highest concentrations tested, despite robust binding (e.g., D4-126 and genotype III) (Figures 5A, 6A, and 6B). Other mAbs have similar neutralization titers despite lower levels of binding (e.g., 5H2 and genotype V). These results reveal that, for each mAb and virus, the amount of mAb sufficient to bind and/or neutralize varies significantly.

Consonant with the binding results, EDE mAbs C10 and B7 potently neutralize all viruses similarly (Figures 6A and 6B), with the exception of B7 and genotype V, due to its missing glycosylation site. The C10 and B7 epitopes are highly conserved across

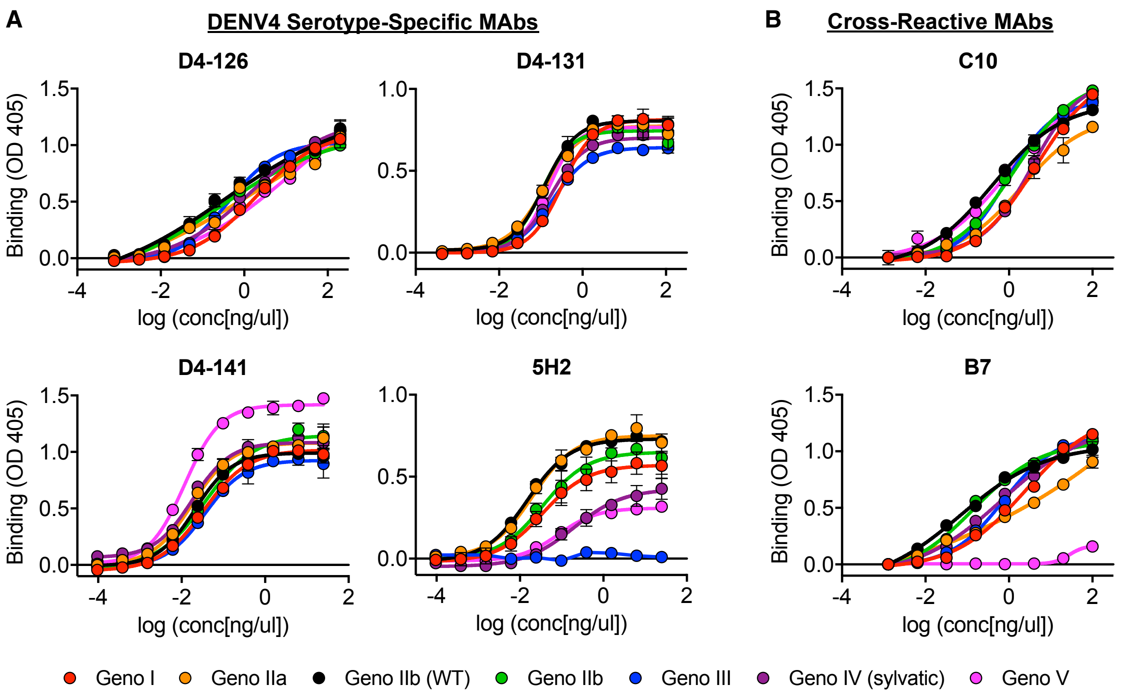

Figure 5. mAbs Differentially Bind DENV4 Genotype Viruses

(A and B) DENV4 serotype-specific antibodies D4-126, D4-131, D4-141, and 5H2 (A) and DENV cross-reactive antibodies C10 and B7 (B) were evaluated for their ability to bind each virus at a range of antibody concentrations using ELISAs (mean ± SD of technical duplicates).

the DENV4 panel, likely explaining the robust and consistent neutralizing titers (Figures S4A and S4B). Additionally, the range of neutralization titers is smaller for cross-reactive mAbs compared to serotype-specific mAbs, suggesting that the more cross-reactive an antibody is (i.e., the more serotypes it recognizes), the less genotypic diversity matters.

The Neutralization of DENV4 E Genotype Variant Viruses by Human Sera from Natural Infection and Vaccination

Convalescent immune sera from people who have recovered from primary DENV4 infections contain strongly neutralizing serotype-specific and weakly neutralizing cross-reactive antibodies. We performed neutralization assays with DENV4 convalescent immune sera to measure the breadth of neutralization across different DENV4 E variant genotypes (Figures 7A and S6A). While the absolute neutralization titers vary across samples by 1–2 logs, all DENV4 immune sera were able to neutralize all genotypes (Figure 7A). While this suggests that natural infection with any DENV4 genotype elicits antibodies that are neutralizing against other genotypes as well, individuals who have weaker responses may be vulnerable to reinfection due to genotype variation.

Individuals who received a genotype II DENV4 monovalent vaccine developed neutralizing antibodies (Figures 7B and S6B). As seen with natural isolates (Durbin et al., 2013), we also observed a larger spread in neutralization titers with the monovalent vaccine immune sera (>2 logs) compared to the natural infection sera. Additionally, for some vaccine sera, neutralizing antibodies were undetectable against some genotypes, despite robust neutralization of other strains (e.g., sample 68 does not neutralize genotype IV or V, but potently neutralizes genotype II viruses). Among the currently circulating genotype I, II, and III viruses, vaccine-matched genotype II viruses were most potently neutralized. To determine whether the differential genotype neutralization is driven by serotype-specific or cross-reactive antibodies, we used depletion techniques to specifically remove cross-reactive antibodies (de Alwis et al., 2012) (Figures 7C, 7D, and S6C–S6E). We find that removing cross-reactive antibodies minimally alters neutralization titers, suggesting that the majority of total neutralization comes from serotype-specific antibodies, and that the differences in titers across DENV4 genotypes are primarily driven by DENV4 serotype-specific antibodies as well.

We next looked at the DENV4 genotypic neutralizing breadth of individuals that received a tetravalent DENV vaccine. As tetravalent vaccination can result in both DENV4 serotype-specific antibodies, and strongly neutralizing cross-reactive antibodies, depletion techniques were again used to determine the contribution of each population of antibodies to total neutralization. Control depleted sera, containing both serotype-specific and cross-reactive antibodies, differentially neutralized the DENV4 variants, with vaccine-matched genotype II viruses neutralized on average 3- to 20-fold more efficiently than the other genotypes (Figures 7E and S7A). In addition, some sera failed to neutralize currently circulating genotype I or III variants. When we removed DENV serotype cross-reactive antibodies, we observed only a small reduction in neutralization titers, indicating that the vaccine mainly induced serotype-specific neutralizing antibodies

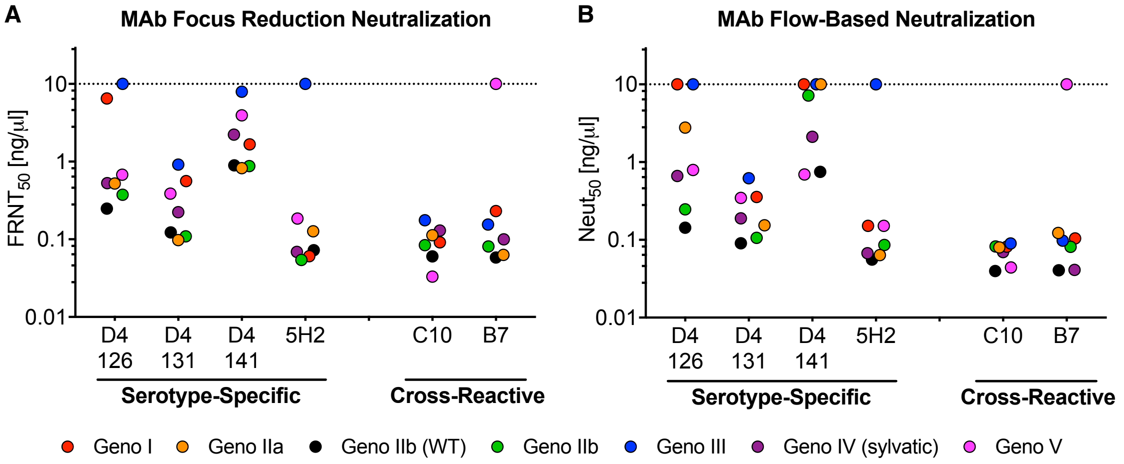

Figure 6. DENV4 Genotypic Variants Are Differentially Neutralized by Monoclonal Antibodies

(A and B) DENV4 serotype-specific antibodies D4-126, D4-131, D4-141, and 5H2 and DENV cross-reactive antibodies C10 and B7 were evaluated for their ability to neutralize DENV4 genotype viruses in (A) Vero cell focus reduction neutralization test (FRNT) and (B) flow cytometry-based neutralization assay (Neut) (mean ± SD of technical duplicates). The y-axes represent the concentration of antibody required to neutralize 50% of infectious virus. The dashed line represents assay limit of detection.

(Figures 7F and S7B). Importantly, after removing cross-reactive antibodies, we see a similar spread in titers across the panel, suggesting that DENV4 serotype-specific antibodies are driving the differential genotypic neutralization. When all DENV cross-reactive and serotype-specific antibodies were depleted, we completely lost neutralization against all viruses (Figure S7C).

DISCUSSION

DENV is the most significant arthropod-borne virus, causing significant morbidity and mortality worldwide. Sanofi-Pasteur's tetravalent DENV vaccine, Dengvaxia, has been marketed and used in human populations, and there are two additional commercial tetravalent vaccine candidates under evaluation in phase III human trials, including the NIH tetravalent DENV vaccine. Recent results with Dengvaxia demonstrate high vaccine efficacy in people who were dengue immune prior to vaccination (81.9%), and much poorer efficacy in people who were naive before vaccination (52.5%) (Hadinegoro et al., 2015). Moreover, naive individuals who received the vaccine appear to be at greater risk of developing severe disease, when exposed to a natural DENV infection approximately 24 months or more following the last dose of vaccine. As a result, Dengvaxia is currently recommended only for use in people who have been primed by natural DENV infections (Sridhar et al., 2018). Dengvaxia stimulated high levels of DENV4 serotype-specific neutralizing antibodies (Henein et al., 2017), and overall vaccine efficacy was highest against DENV4. However, in subjects who experienced DENV4 breakthrough infections, molecular analyses indicated that the vaccine had a greater efficacy against vaccine-matched DENV4 genotype II than the co-circulating genotype I virus (Rabaa et al., 2017). These data underscore the need for developing viruses and reagents that capture intra-serotype genetic variation when evaluating vaccine immune responses and identifying potential antibody-based correlates of protective immunity.

The existence of phylogenetically and antigenically distinct DENV1–DENV4 serotypes is well accepted in the literature (Holmes and Twiddy, 2003); however, the role of genetic diversity across genotypes is less well studied. Many common laboratory DENV strains have either been heavily cell culture adapted and/or differ in sequence from contemporary circulating strains (Dowd et al., 2015; Katzelnick et al., 2015, 2017). Additionally, some laboratory, and importantly, vaccine strains, are composed of DENV genotypes that are likely extinct and, consequently, do not circulate in human populations (Katzelnick et al., 2017). While $CD8^+$ T cells, $CD4^+$ T cells, and other mechanisms of cellular immunity are correlated with DENV protective immunity (Mathew and Rothman, 2008), and antibodies against DENV NS1 may also alter disease severity (Hertz et al., 2017), neutralizing antibodies represent the best correlate of protection to date (Katzelnick et al., 2016; Buddhari et al., 2014).

Natural DENV infection is thought to provide lifelong protection against symptomatic reinfection with that serotype (Katzelnick et al., 2016; Buddhari et al., 2014); however, it is unknown whether individuals are protected with the same efficacy against all genotypes within the serotype. There are reports of rare, typically asymptomatic, homotypic reinfection in people in Nicaragua and Peru (Forshey et al., 2016; Waggoner et al., 2016), which is potentially driven by genotypic differences between the primary and secondary infecting viruses. Some studies have evaluated the breadth of antibody neutralization against different genotypes elicited by natural infection or vaccination (Blaney et al., 2005; Durbin et al., 2013; Katzelnick et al., 2015; Messer et al., 2012; Vasilakis et al., 2008a). While most individuals exposed to natural infections or vaccines neutralized multiple genotypes within each serotype, absolute levels of neutralizing antibodies vary depending on the individual and the DENV genotypes used. Indeed, even in the current study, we noted that most individuals exposed to natural infections or a vaccine, developed antibodies that neutralized the most

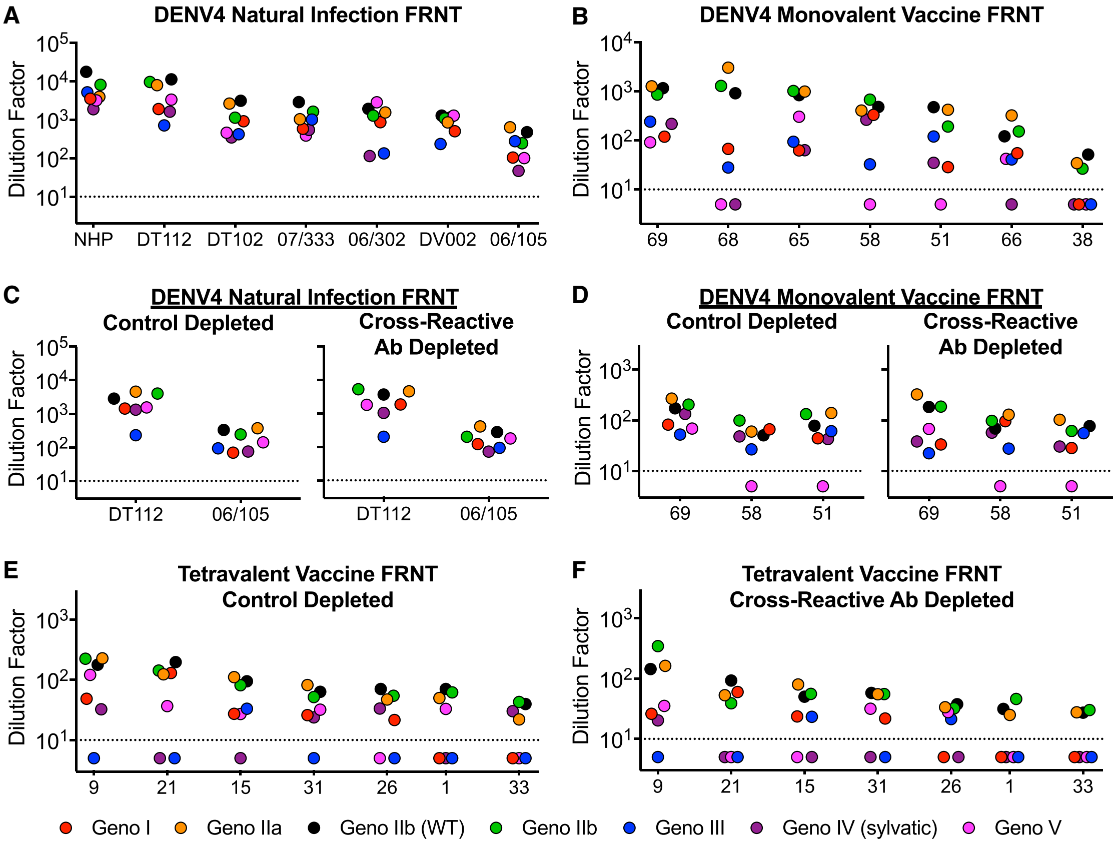

Figure 7. DENV4 Polyclonal Immune Sera Have a Range of Neutralization Titers against DENV4 Variants Driven by Serotype-Specific Antibodies

(A and B) Using Vero cell focus reduction neutralization test (FRNT), (A) pooled polyclonal immune sera from DENV4-infected non-human primates (NHPs) or from naturally infected individuals, or from (B) individuals who received the NIH DENV4 monovalent vaccine were evaluated for their ability to neutralize DENV4 genotype viruses.

(C–F) DENV4 natural infection sera (C), DENV4 monovalent vaccine sera (D), and NIH DENV tetravalent vaccine sera were control depleted with BSA (E), or depleted of cross-reactive antibodies (F) and evaluated for their ability to neutralize DENV4 genotype viruses. The y axis represents the dilution factor of immune sera required to neutralize 50% of infectious virus (mean ± SD of technical duplicates). The dashed line represents one-half the assay limit of detection.

prevalent DENV4 genotypes, but the levels of neutralizing antibody varied considerably by genotype. In 2008, the World Health Organization (WHO) noted that there is little evidence of antigenic drift within DENV serotypes that might lead to resistance of certain strains to post-vaccination neutralization, yet they advised that laboratories consider inclusion of multiple virus strains, including laboratory prototype strains and recent clinical isolates when performing neutralization assays (Roehrig et al., 2008). Our results demonstrate the value of including different DENV genotypes when evaluating vaccine responses.

Using reverse genetics, we developed an isogenic panel of DENV4 recombinant viruses that only differ in their E glycoprotein, which was derived from different genotypes. We reconstructed clinically relevant isolates with E protein genes derived from clinical specimens or low passage history in culture. Our data demonstrate that DENV4 E protein genotypic diversity can impact many aspects of the virus's cell biology including growth in cells, glycosylation, syncytial formation, and maturation. Additionally, as all the viruses can be enhanced, it highlights the importance of determining the impact of DENV genetic variation on disease enhancement after infection and vaccination. The chimeric DENV4 virus panel described here is a powerful tool for initial assessment of the impact of DENV E genotypic variation on virus biology and humoral immunity. As we selected one representative envelope sequence per genotype and utilized a recombinant approach, the viruses here do not capture all E protein sequence diversity within each genotype and some will never actually be encountered by vaccine recipients. Hence, it will be important to evaluate more contemporary DENV4 genotype I, II, and III strains in future studies, including the use of both natural and recombinantly derived isolates. In agreement with our findings, we note that another study using WT strains of endemic and sylvatic DENV4 viruses demonstrated better neutralization of vaccine-matched endemic genotype II viruses compared to sylvatic viruses using sera from DENV4 monovalent vaccine recipients (Durbin et al., 2013).

Our results demonstrate that infection or vaccination with a single DENV4 genotype stimulates variable levels of neutralizing

antibodies to different genotypes. Currently, there are insufficient data to correlate levels of neutralizing antibodies to protection from DENV disease. Moreover, other immune mechanisms involving T cells, NS1 immunity, and B cell memory may also reduce or eliminate clinical disease (Mathew and Rothman, 2008). However, it is worth noting that the licensed tetravalent DENV vaccine (Dengvaxia) had higher vaccine efficacy against vaccine-matched DENV4 genotype II (~83%) viruses compared to co-circulating DENV4 genotype I viruses (~47%) (Rabaa et al., 2017). We propose that targeted surveillance of changing or emerging DENV genotypes following vaccination will be valuable in assessing the influence of DENV genotype on the frequency of repeat infections and overall vaccine effectiveness.

EXPERIMENTAL PROCEDURES

Phylogenetic Tree

The tree was constructed in Geneious R11 using the neighbor-joining method (Jukes–Cantor genetic distance) with 100 replicates based on the multiple sequence alignment. The radial phylogram was visualized and rendered for publication using CLC Sequence Viewer 7 and Adobe Illustrator CC 2017.

Virus Construction

Chimeric recombinant DENV4 viruses were constructed as described before (Gallichotte et al., 2015, 2017). Briefly, DNA encoding isogenic envelope protein sequences was introduced into a quadripartite DENV4 infectious clone system using synthetically derived genes and recombinant DNA approaches. Plasmid DNA was digested and ligated together, and viral full-length genomic RNA was generated using T7 RNA polymerase. Infectious genome-length capped viral RNA transcripts were electroporated into C6/36 cells, and supernatant was harvested and passaged onto C6/36 cells to make viral working stocks.

Cells

C6/36 cells (ATCC CRL-1660) were grown in minimum essential medium (MEM) at 32°C. Vero cells (ATCC CCL-81) were grown in DMEM media, U937 and U937 cells stably expressing DC-SIGN (U937+DC-SIGN) were grown in RPMI medium 1640, and all were cultured at 37°C. All media were supplemented with 5% fetal bovine serum (FBS), which was reduced to 2% during DENV infection. All media were supplemented with 100 U/mL penicillin, 100 μg/mL streptomycin, and 0.25 μg/mL amphotericin B. C6/36 and U937/U937+DC-SIGN media were additionally supplemented with non-essential amino acids, and U937/U937+DC-SIGN media was further supplemented with L-glutamine and β-mercaptoethanol. All cells were incubated at 5% CO_2.

Immune Sera

DENV4 immune non-human primate serum was obtained from BEI Resources (NR-41789). Human dengue immune sera were obtained from a previously described Dengue Traveler collection at the University of North Carolina. Vaccine sera were obtained from individuals who received a live-attenuated monovalent DENV4 or tetravalent vaccine 180 days post-vaccination, as developed by the NIH, and were provided by A.P.D. and S.S.W. All human sera samples were anonymized and obtained under Institutional Review Board approval.

Viral Titering and Immunostaining

Cells were plated 1 day prior to infection. Growth media was removed, and virus stocks were serially diluted 10-fold, added to cells, and incubated for 1 hr at either 32°C (C6/36) or 37°C (Vero). After incubation, 1% methylcellulose in Opti-MEM (supplemented with 2% FBS, 100 U/mL penicillin, 100 μg/mL streptomycin, and 0.25 μg/mL amphotericin B) was overlaid, and cells were incubated for 3–4 days. Cells were washed with PBS and fixed with 80% methanol. Cells were blocked in 5% non-fat dried milk and stained with anti-E (4G2) and anti-prM (2H2) mAbs and horseradish peroxidase (HRP)-labeled secondary antibody. Foci were developed using TrueBlue substrate, and viral foci were counted manually.

Growth Curves

C6/36 or Vero cells were seeded in 24-well plates 1 day prior to infection. Viruses were diluted to an MOI of either 0.01 or 0.5, added to cells, and incubated for 1 hr at either 32°C (C6/36) or 37°C (Vero). Inoculum was removed, cells were washed three times with PBS, and growth media were replaced. Media were sampled daily, replaced with fresh media, and immediately frozen at −80°C. Samples were titered as described above.

Thermostability Assay

DENV4 viruses were diluted 1:10, then incubated at 4°C, 28°C, 37°C, or 40°C for 1 hr, then immediately transferred to 4°C for 15 min. Viruses were then titered on Vero cells and immunostained as described above.

Immunoblotting

Virus stocks were diluted in PBS, mixed with sample buffer, and heated at 95°C for 10 min. Samples were run on 4%–20% Protean TGX gels and transferred to polyvinylidene difluoride (PVDF) membrane. Membranes were blocked in 5% non-fat dried milk and probed with anti-E (4G2) and anti-prM (1E16) mAbs. Membranes were washed and probed with secondary antibodies labeled with HRP and developed using chemiluminescent substrate. Membranes were visualized using a LI-COR C-DiGit Blot Scanner.

Enzyme-Linked Immunosorbent Binding Assay

Plates were coated with anti-E (4G2) and anti-prM (2H2) antibodies in carbonate buffer overnight and blocked in 5% non-fat dried milk, and then virus antigen was added. Primary antibody was diluted in blocking buffer and added to plates for 1 hr at 37°C. Alkaline-phosphate-labeled secondary antibody was added and plates were incubated for 1 hr at 37°C. Plates were developed with p-nitrophenyl phosphate substrate and color changes were quantified using Bio-Rad iMark Microplate Absorbance Reader.

ADE Assay

mAbs was diluted 5-fold and mixed with virus previously diluted to result in ~15% infection in U937+DC-SIGN cells. Virus:mAb mixtures were incubated at 37°C for 45 min, and then added to 5 × 10^4 U937 cells and incubated at 37°C for 2 hr. After incubation, cells were washed with growth media, and then resuspended in fresh growth media. The cells were incubated for 20 hr at 37°C, washed in PBS, fixed in 10% phosphate-buffered formalin, and then stained with anti-E mAb 4G2 directly conjugated to Alexa Fluor 488. Cells were analyzed on a Guava easyCyte flow cytometer.

Neutralization Assays

FRNT was performed by seeding Vero cells 1 day prior to infection. mAbs or immune sera were diluted 4-fold and mixed with virus stocks previously diluted to ~40 ffu/well. Virus:Ab mixtures were incubated at 37°C for 1 hr, and then added to cells for 1 hr at 37°C. After incubation, overlay media was added and plates were incubated for 3 days. Cells were fixed and immunostained as described as above. Flow cytometry-based neutralization assays were performed as described above in ADE assays, except with U937+DC-SIGN cells.

Polyclonal Antibody Depletion Assay

Dynabeads were covalently bound to anti-E mAb 1M7 overnight at 37°C. Bead:mAb complex was blocked with 1% BSA in PBS at 37°C, and then washed with 0.1 M 2-(*N*-morpholino)ethanesulfonic acid (MES) buffer. Beads were incubated with BSA (control), purified DENV3 (cross-reactive depletion), or a mix of DENV3 and DENV4 (full depletion) for 1 hr at 37°C, and then washed three times with PBS. Bead:mAb:DENV complex was fixed with 2% paraformaldehyde in PBS for 20 min, and then washed four times with PBS. DENV-specific antibodies were depleted from sera by incubating beads with sera diluted 1:10 in PBS for 1 hr at 37°C with end-over-end mixing for at least two sequential rounds of depletions. Removal of DENV antibodies was confirmed by ELISA.

Data Analysis and Software

All data were analyzed and graphed using GraphPad Prism v7.0a. Protein structures were visualized using MacPyMOL: PyMOL v1.7.6.2. Replicate information is included in the figure legends.

SUPPLEMENTAL INFORMATION

Supplemental Information includes seven figures and three tables and can be found with this article online at https://doi.org/10.1016/j.celrep.2018.10.006.

ACKNOWLEDGMENTS

This research was supported by National Institute of Allergy and Infectious Diseases (NIAID) grants R01s AI107731 (principal investigator [PI], A.M.d.S.) and AI125198 (PI, A.M.d.S.), P01 AI106695 (PI, E. Harris), and U19 AI109761 (PI, R.S.B.) and by grant OPP1104710 from the Bill and Melinda Gates Foundation (PI, A.P.D.). Support was also provided in part by the Intramural Research Program of the NIAID. E.N.G. was supported by T32 NIH Training Grant AI007419. We thank Stephanie Marcet, Scott Royal, and Prem Lakshmanane for assistance.

AUTHOR CONTRIBUTIONS

Conceptualization, E.N.G., A.M.d.S., and R.S.B.; Investigation, E.N.G., T.J.B, U.N., M.J.D., R.G., D.G.W., and B.L.Y.; Resources, A.P.D., S.S.W., A.M.d.S., and R.S.B.; Writing – Original Draft, E.N.G.; Writing – Review & Editing, E.N.G., A.P.D., S.S.W., A.M.d.S., and R.S.B.; Supervision, A.M.d.S. and R.S.B.

DECLARATION OF INTERESTS

A.M.d.S. has consulted on dengue vaccines for Takeda, Merck, and GSK. R.S.B. has consulted with Takeda on vaccines. A.M.d.S. and R.S.B. are inventors on patents filed and issues relevant to dengue vaccines.

Received: June 11, 2018
Revised: August 15, 2018
Accepted: September 28, 2018
Published: October 30, 2018

REFERENCES

Bennett, S.N., Drummond, A.J., Kapan, D.D., Suchard, M.A., Muñoz-Jordán, J.L., Pybus, O.G., Holmes, E.C., and Gubler, D.J. (2010). Epidemic dynamics revealed in dengue evolution. Mol. Biol. Evol. *27*, 811–818.

Bhatt, S., Gething, P.W., Brady, O.J., Messina, J.P., Farlow, A.W., Moyes, C.L., Drake, J.M., Brownstein, J.S., Hoen, A.G., Sankoh, O., et al. (2013). The global distribution and burden of dengue. Nature *496*, 504–507.

Blaney, J.E., Jr., Matro, J.M., Murphy, B.R., and Whitehead, S.S. (2005). Recombinant, live-attenuated tetravalent dengue virus vaccine formulations induce a balanced, broad, and protective neutralizing antibody response against each of the four serotypes in rhesus monkeys. J. Virol. *79*, 5516–5528.

Bryant, J.E., Calvert, A.E., Mesesan, K., Crabtree, M.B., Volpe, K.E., Silengo, S., Kinney, R.M., Huang, C.Y., Miller, B.R., and Roehrig, J.T. (2007). Glycosylation of the dengue 2 virus E protein at N67 is critical for virus growth in vitro but not for growth in intrathoracically inoculated *Aedes aegypti* mosquitoes. Virology *366*, 415–423.

Buddhari, D., Aldstadt, J., Endy, T.P., Srikiatkhachorn, A., Thaisomboonsuk, B., Klungthong, C., Nisalak, A., Khuntirat, B., Jarman, R.G., Fernandez, S., et al. (2014). Dengue virus neutralizing antibody levels associated with protection from infection in thai cluster studies. PLoS Negl. Trop. Dis. *8*, e3230.

Calisher, C.H., Karabatsos, N., Dalrymple, J.M., Shope, R.E., Porterfield, J.S., Westaway, E.G., and Brandt, W.E. (1989). Antigenic relationships between flaviviruses as determined by cross-neutralization tests with polyclonal antisera. J. Gen. Virol. *70*, 37–43.

Cao-Lormeau, V.M., Roche, C., Aubry, M., Teissier, A., Lastere, S., Daudens, E., Mallet, H.P., Musso, D., and Aaskov, J. (2011). Recent emergence of dengue virus serotype 4 in French Polynesia results from multiple introductions from other South Pacific Islands. PLoS One *6*, e29555.

Chen, R., and Han, G.Z. (2016). Dengue in China: comprehensive phylogenetic evaluation reveals evidence of endemicity and complex genetic diversity. Am. J. Trop. Med. Hyg. *94*, 198–202.

Cockburn, J.J., Navarro Sanchez, M.E., Goncalvez, A.P., Zaitseva, E., Stura, E.A., Kikuti, C.M., Duquerroy, S., Dussart, P., Chernomordik, L.V., Lai, C.J., and Rey, F.A. (2012). Structural insights into the neutralization mechanism of a higher primate antibody against dengue virus. EMBO J. *31*, 767–779.

Coloma, J., and Harris, E. (2015). Broad and strong: the ultimate antibody to dengue virus. Nat. Immunol. *16*, 135–137.

Dash, P.K., Sharma, S., Srivastava, A., Santhosh, S.R., Parida, M.M., Neeraja, M., Subbalaxmi, M.V., Lakshmi, V., and Rao, P.V. (2011). Emergence of dengue virus type 4 (genotype I) in India. Epidemiol. Infect. *139*, 857–861.

de Alwis, R., Smith, S.A., Olivarez, N.P., Messer, W.B., Huynh, J.P., Wahala, W.M., White, L.J., Diamond, M.S., Baric, R.S., Crowe, J.E., Jr., and de Silva, A.M. (2012). Identification of human neutralizing antibodies that bind to complex epitopes on dengue virions. Proc. Natl. Acad. Sci. USA *109*, 7439–7444.

Dowd, K.A., DeMaso, C.R., and Pierson, T.C. (2015). Genotypic differences in dengue virus neutralization are explained by a single amino acid mutation that modulates virus breathing. MBio *6*, e01559-15.

Durbin, A.P., Mayer, S.V., Rossi, S.L., Amaya-Larios, I.Y., Ramos-Castaneda, J., Eong Ooi, E., Jane Cardosa, M., Munoz-Jordan, J.L., Tesh, R.B., Messer, W.B., et al. (2013). Emergence potential of sylvatic dengue virus type 4 in the urban transmission cycle is restrained by vaccination and homotypic immunity. Virology *439*, 34–41.

Fares, R.C., Souza, K.P., Añez, G., and Rios, M. (2015). Epidemiological scenario of dengue in Brazil. BioMed Res. Int. *2015*, 321873.

Fleith, R.C., Lobo, F.P., Dos Santos, P.F., Rocha, M.M., Bordignon, J., Strottmann, D.M., Patricio, D.O., Pavanelli, W.R., Lo Sarzi, M., Santos, C.N., et al. (2016). Genome-wide analyses reveal a highly conserved dengue virus envelope peptide which is critical for virus viability and antigenic in humans. Sci. Rep. *6*, 36339.

Forshey, B.M., Reiner, R.C., Olkowski, S., Morrison, A.C., Espinoza, A., Long, K.C., Vilcarromero, S., Casanova, W., Wearing, H.J., Halsey, E.S., et al. (2016). Incomplete protection against dengue virus type 2 re-infection in Peru. PLoS Negl. Trop. Dis. *10*, e0004398.

Gallichotte, E.N., Widman, D.G., Yount, B.L., Wahala, W.M., Durbin, A., Whitehead, S., Sariol, C.A., Crowe, J.E., Jr., de Silva, A.M., and Baric, R.S. (2015). A new quaternary structure epitope on dengue virus serotype 2 is the target of durable type-specific neutralizing antibodies. MBio *6*, e01461-15.

Gallichotte, E.N., Menachery, V.D., Yount, B.L., Jr., Widman, D.G., Dinnon, K.H., 3rd, Hartman, S., de Silva, A.M., and Baric, R.S. (2017). Epitope addition and ablation via manipulation of a dengue virus serotype 1 infectious clone. MSphere *2*, e00380-16.

Hadinegoro, S.R., Arredondo-García, J.L., Capeding, M.R., Deseda, C., Chotpitayasunondh, T., Dietze, R., Muhammad Ismail, H.I., Reynales, H., Limkittikul, K., Rivera-Medina, D.M., et al.; CYD-TDV Dengue Vaccine Working Group (2015). Efficacy and long-term safety of a dengue vaccine in regions of endemic disease. N. Engl. J. Med. *373*, 1195–1206.

Halstead, S.B. (2015). Pathogenesis of dengue: dawn of a new era. F1000Res. *4*, F1000 Faculty Rev-1353.

Henein, S., Swanstrom, J., Byers, A.M., Moser, J.M., Shaik, S.F., Bonaparte, M., Jackson, N., Guy, B., Baric, R., and de Silva, A.M. (2017). Dissecting antibodies induced by a chimeric yellow fever-dengue, live-attenuated, tetravalent dengue vaccine (CYD-TDV) in naive and dengue-exposed individuals. J. Infect. Dis. *215*, 351–358.

Hertz, T., Beatty, P.R., MacMillen, Z., Killingbeck, S.S., Wang, C., and Harris, E. (2017). Antibody epitopes identified in critical regions of dengue virus nonstructural 1 protein in mouse vaccination and natural human infections. J. Immunol. *198*, 4025–4035.

Holmes, E.C., and Twiddy, S.S. (2003). The origin, emergence and evolutionary genetics of dengue virus. Infect. Genet. Evol. *3*, 19–28.

Katzelnick, L.C., Fonville, J.M., Gromowski, G.D., Bustos Arriaga, J., Green, A., James, S.L., Lau, L., Montoya, M., Wang, C., VanBlargan, L.A., et al.

(2015). Dengue viruses cluster antigenically but not as discrete serotypes. Science *349*, 1338–1343.

Katzelnick, L.C., Montoya, M., Gresh, L., Balmaseda, A., and Harris, E. (2016). Neutralizing antibody titers against dengue virus correlate with protection from symptomatic infection in a longitudinal cohort. Proc. Natl. Acad. Sci. USA *113*, 728–733.

Katzelnick, L.C., and Harris, E.; Participants in the Summit on Dengue Immune Correlates of Protection (2017). Immune correlates of protection for dengue: state of the art and research agenda. Vaccine *35*, 4659–4669.

Klungthong, C., Zhang, C., Mammen, M.P., Jr., Ubol, S., and Holmes, E.C. (2004). The molecular epidemiology of dengue virus serotype 4 in Bangkok, Thailand. Virology *329*, 168–179.

Lee, E., Leang, S.K., Davidson, A., and Lobigs, M. (2010). Both E protein glycans adversely affect dengue virus infectivity but are beneficial for virion release. J. Virol. *84*, 5171–5180.

Lim, X.X., Chandramohan, A., Lim, X.E., Crowe, J.E., Jr., Lok, S.M., and Anand, G.S. (2017). Epitope and paratope mapping reveals temperature-dependent alterations in the dengue-antibody interface. Structure *25*, 1391–1402.e3.

Mathew, A., and Rothman, A.L. (2008). Understanding the contribution of cellular immunity to dengue disease pathogenesis. Immunol. Rev. *225*, 300–313.

Messer, W.B., Yount, B., Hacker, K.E., Donaldson, E.F., Huynh, J.P., de Silva, A.M., and Baric, R.S. (2012). Development and characterization of a reverse genetic system for studying dengue virus serotype 3 strain variation and neutralization. PLoS Negl. Trop. Dis. *6*, e1486.

Messina, J.P., Brady, O.J., Scott, T.W., Zou, C., Pigott, D.M., Duda, K.A., Bhatt, S., Katzelnick, L., Howes, R.E., Battle, K.E., et al. (2014). Global spread of dengue virus types: mapping the 70 year history. Trends Microbiol. *22*, 138–146.

Mondotte, J.A., Lozach, P.Y., Amara, A., and Gamarnik, A.V. (2007). Essential role of dengue virus envelope protein N glycosylation at asparagine-67 during viral propagation. J. Virol. *81*, 7136–7148.

Mukherjee, S., Dowd, K.A., Manhart, C.J., Ledgerwood, J.E., Durbin, A.P., Whitehead, S.S., and Pierson, T.C. (2014). Mechanism and significance of cell type-dependent neutralization of flaviviruses. J. Virol. *88*, 7210–7220.

Nivarthi, U.K., Kose, N., Sapparapu, G., Widman, D., Gallichotte, E., Pfaff, J.M., Doranz, B.J., Weiskopf, D., Sette, A., Durbin, A.P., et al. (2017). Mapping the human memory B cell and serum neutralizing antibody responses to dengue virus serotype 4 infection and vaccination. J. Virol. *91*, e02041-16.

Pierro, D.J., Salazar, M.I., Beaty, B.J., and Olson, K.E. (2006). Infectious clone construction of dengue virus type 2, strain Jamaican 1409, and characterization of a conditional E6 mutation. J. Gen. Virol. *87*, 2263–2268.

Pierson, T.C., and Diamond, M.S. (2012). Degrees of maturity: the complex structure and biology of flaviviruses. Curr. Opin. Virol. *2*, 168–175.

Pokidysheva, E., Zhang, Y., Battisti, A.J., Bator-Kelly, C.M., Chipman, P.R., Xiao, C., Gregorio, G.G., Hendrickson, W.A., Kuhn, R.J., and Rossmann, M.G. (2006). Cryo-EM reconstruction of dengue virus in complex with the carbohydrate recognition domain of DC-SIGN. Cell *124*, 485–493.

Rabaa, M.A., Girerd-Chambaz, Y., Duong Thi Hue, K., Vu Tuan, T., Wills, B., Bonaparte, M., van der Vliet, D., Langevin, E., Cortes, M., Zambrano, B., et al. (2017). Genetic epidemiology of dengue viruses in phase III trials of the CYD tetravalent dengue vaccine and implications for efficacy. eLife *6*, e24196.

Rey, F.A., Stiasny, K., Vaney, M.C., Dellarole, M., and Heinz, F.X. (2018). The bright and the dark side of human antibody responses to flaviviruses: lessons for vaccine design. EMBO Rep. *19*, 206–224.

Roehrig, J.T., Hombach, J., and Barrett, A.D. (2008). Guidelines for plaque-reduction neutralization testing of human antibodies to dengue viruses. Viral Immunol. *21*, 123–132.

Rossi, S.L., Nasar, F., Cardosa, J., Mayer, S.V., Tesh, R.B., Hanley, K.A., Weaver, S.C., and Vasilakis, N. (2012). Genetic and phenotypic characterization of sylvatic dengue virus type 4 strains. Virology *423*, 58–67.

Rouvinski, A., Guardado-Calvo, P., Barba-Spaeth, G., Duquerroy, S., Vaney, M.C., Kikuti, C.M., Navarro Sanchez, M.E., Dejnirattisai, W., Wongwiwat, W., Haouz, A., et al. (2015). Recognition determinants of broadly neutralizing human antibodies against dengue viruses. Nature *520*, 109–113.

Shihada, S., Emmerich, P., Thomé-Bolduan, C., Jansen, S., Günther, S., Frank, C., Schmidt-Chanasit, J., and Cadar, D. (2017). Genetic diversity and new lineages of dengue virus serotypes 3 and 4 in returning travelers, Germany, 2006–2015. Emerg. Infect. Dis. *23*, 272–275.

Smith, S.A., Nivarthi, U.K., de Alwis, R., Kose, N., Sapparapu, G., Bombardi, R., Kahle, K.M., Pfaff, J.M., Lieberman, S., Doranz, B.J., et al. (2015). Dengue virus prM-specific human monoclonal antibodies with virus replication-enhancing properties recognize a single immunodominant antigenic site. J. Virol. *90*, 780–789.

Sridhar, S., Luedtke, A., Langevin, E., Zhu, M., Bonaparte, M., Machabert, T., Savarino, S., Zambrano, B., Moureau, A., Khromava, A., et al. (2018). Effect of dengue serostatus on dengue vaccine safety and efficacy. N. Engl. J. Med. *379*, 327–340.

Teoh, B.T., Sam, S.S., Abd-Jamil, J., and AbuBakar, S. (2010). Isolation of ancestral sylvatic dengue virus type 1, Malaysia. Emerg. Infect. Dis. *16*, 1783–1785.

Vasilakis, N., Durbin, A.P., da Rosa, A.P., Munoz-Jordan, J.L., Tesh, R.B., and Weaver, S.C. (2008a). Antigenic relationships between sylvatic and endemic dengue viruses. Am. J. Trop. Med. Hyg. *79*, 128–132.

Vasilakis, N., Tesh, R.B., and Weaver, S.C. (2008b). Sylvatic dengue virus type 2 activity in humans, Nigeria, 1966. Emerg. Infect. Dis. *14*, 502–504.

Waggoner, J.J., Balmaseda, A., Gresh, L., Sahoo, M.K., Montoya, M., Wang, C., Abeynayake, J., Kuan, G., Pinsky, B.A., and Harris, E. (2016). Homotypic dengue virus reinfections in Nicaraguan children. J. Infect. Dis. *214*, 986–993.

Weaver, S.C., and Vasilakis, N. (2009). Molecular evolution of dengue viruses: contributions of phylogenetics to understanding the history and epidemiology of the preeminent arboviral disease. Infect. Genet. Evol. *9*, 523–540.

Zhao, R., Chinnawirotpisan, P., Klungthong, C., Zhang, C., and Putnak, R. (2010). Evidence for inter- and intra-genotypic variations in dengue serotype 4 viruses representing predominant and non-predominant genotypes co-circulating in Thailand from 1977 to 2001. Virus Genes *41*, 5–13.

Cell Reports, Volume *25*

Supplemental Information

Genetic Variation between Dengue Virus Type 4 Strains Impacts Human Antibody Binding and Neutralization

Emily N. Gallichotte, Thomas J. Baric, Usha Nivarthi, Matthew J. Delacruz, Rachel Graham, Douglas G. Widman, Boyd L. Yount, Anna P. Durbin, Stephen S. Whitehead, Aravinda M. de Silva, and Ralph S. Baric

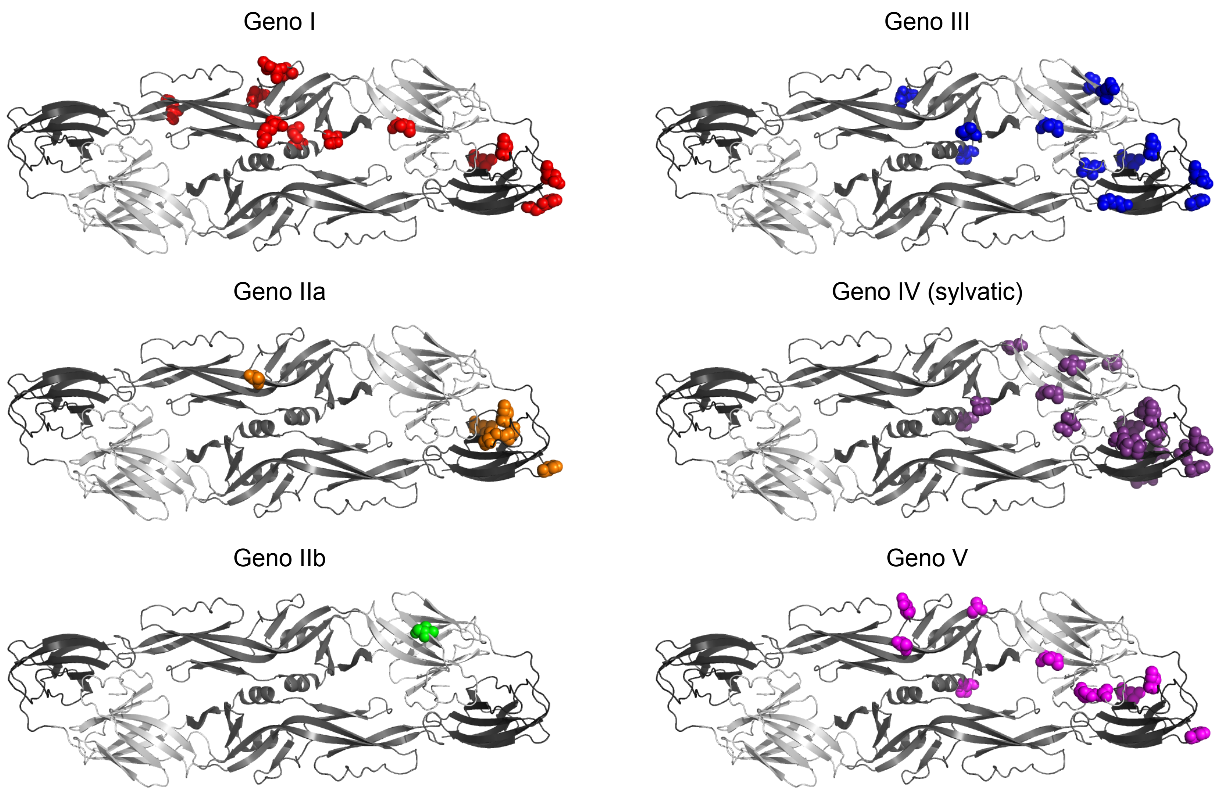

Supplemental Figure 1. Amino acid diversity of each DENV4 genotype virus. Related to Figure 2. Differences in envelope protein amino acid sequence of each genotype relative to WT (genotype IIb) were mapped on envelope protein dimer (PBD = 1OAN).

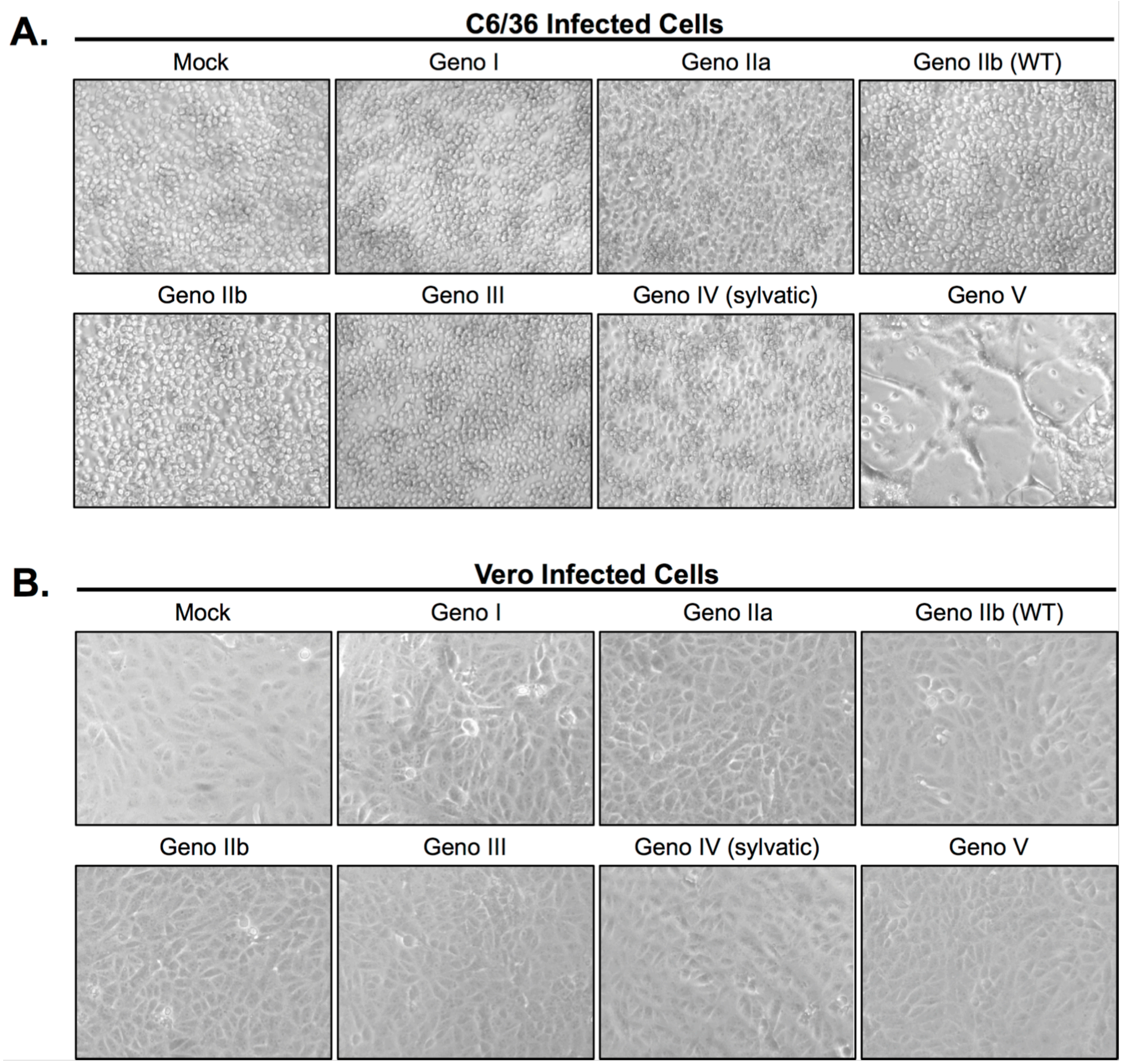

Supplemental Figure 2. Cytopathic effect of cells infected with DENV4 viruses. Related to Figure 3. A) C6/36 and B) Vero cells were infected at a multiplicity of infection (MOI) of 0.5 and imaged four days post-infection.

Virus	1	2	3	4	5	6	7	8	9	10	11	12	13	14	15	16	17	18	19	20	21	22	23	24	25	26	27	28	29	30	31	32	33	34	35	36	37	38	39	40	41	42	43	44	45	46
Geno I	F	H	L	S	T	R	D	G	E	P	L	M	I	V	A	K	H	E	R	G	R	P	L	L	F	K	T	T	E	G	I	N	K	C	T	L	I	A	M	D	L	G	E	M	C	E
Geno IIa*	F	H	L	S	T	R	D	G	E	P	L	M	I	V	A	K	H	E	R	G	R	P	L	L	F	K	T	T	E	G	I	N	K	C	T	L	I	A	M	D	L	G	E	M	C	E
Geno IIb (WT)	F	H	L	S	T	R	D	G	E	P	L	M	I	V	A	K	H	E	R	G	R	P	L	L	F	K	T	T	E	G	I	N	K	C	T	L	I	A	M	D	L	G	E	M	C	E
Geno IIb	F	H	L	S	T	R	D	G	E	P	L	M	I	V	A	K	H	E	R	G	R	P	L	L	F	K	T	T	E	G	I	N	K	C	T	L	I	A	M	D	L	G	E	M	C	E
Geno III*	F	H	L	S	T	R	D	G	E	P	L	M	I	V	A	K	H	E	R	G	R	P	L	L	F	K	T	T	E	G	I	N	K	C	T	L	I	A	M	D	L	G	E	M	C	E
Geno IV (sylvatic)	F	H	L	S	S	R	D	G	E	P	L	M	I	V	A	K	H	E	R	G	R	P	L	L	F	K	T	T	E	G	I	N	K	C	T	L	I	A	M	D	V	G	E	M	C	E
Geno V	F	H	L	S	T	R	D	G	E	P	L	M	I	V	A	K	H	E	R	G	R	P	L	L	F	K	T	T	E	G	I	N	K	C	T	L	I	A	M	D	L	G	E	M	C	E

Virus	47	48	49	50	51	52	53	54	55	56	57	58	59	60	61	62	63	64	65	66	67	68	69	70	71	72	73	74	75	76	77	78	79	80	81	82	83	84	85	86	87	88	89	90	91
Geno I	D	T	V	T	Y	K	C	P	L	L	V	N	T	E	P	E	D	I	D	C	W	C	N	L	T	S	A	W	V	M	Y	G	T	C	T	Q	S	G	E	R	R	R	E	K	R
Geno IIa*	D	T	V	T	Y	K	C	P	L	L	V	N	T	E	P	E	D	I	D	C	W	C	N	L	T	S	T	W	V	M	Y	G	T	C	T	Q	S	G	E	R	R	R	E	K	R
Geno IIb (WT)	D	T	V	T	Y	K	C	P	L	L	V	N	T	E	P	E	D	I	D	C	W	C	N	L	T	S	T	W	V	M	Y	G	T	C	T	Q	S	G	E	R	R	R	E	K	R
Geno IIb	D	T	V	T	Y	K	C	P	L	L	V	N	T	E	P	E	D	I	D	C	W	C	N	L	T	S	T	W	V	M	Y	G	T	C	T	Q	S	G	E	R	R	R	E	K	R
Geno III*	D	T	V	T	Y	K	C	P	L	L	V	N	T	E	P	E	D	I	D	C	W	C	N	L	T	S	T	W	V	M	Y	G	T	C	T	Q	N	G	E	R	R	R	E	K	R
Geno IV (sylvatic)	D	T	V	T	Y	K	C	P	L	L	V	N	T	E	P	E	D	I	D	C	W	C	N	S	T	S	T	W	V	T	Y	G	T	C	T	Q	S	G	E	R	R	R	E	K	R
Geno V	D	T	V	M	Y	K	C	P	L	L	V	N	T	E	P	E	D	I	D	C	W	C	N	L	T	S	T	W	V	M	Y	G	T	C	T	Q	S	G	E	R	R	R	E	K	R

Supplemental Figure 3. Sequence alignment of pr protein. Related to Figure 4. For the genotype IIa and III (*) envelope sequences used in our panel, paired pr sequences were not are available. Consensus pr sequences based on those available from other viruses within genotypes IIa and III in the phylogenetic tree are shown.

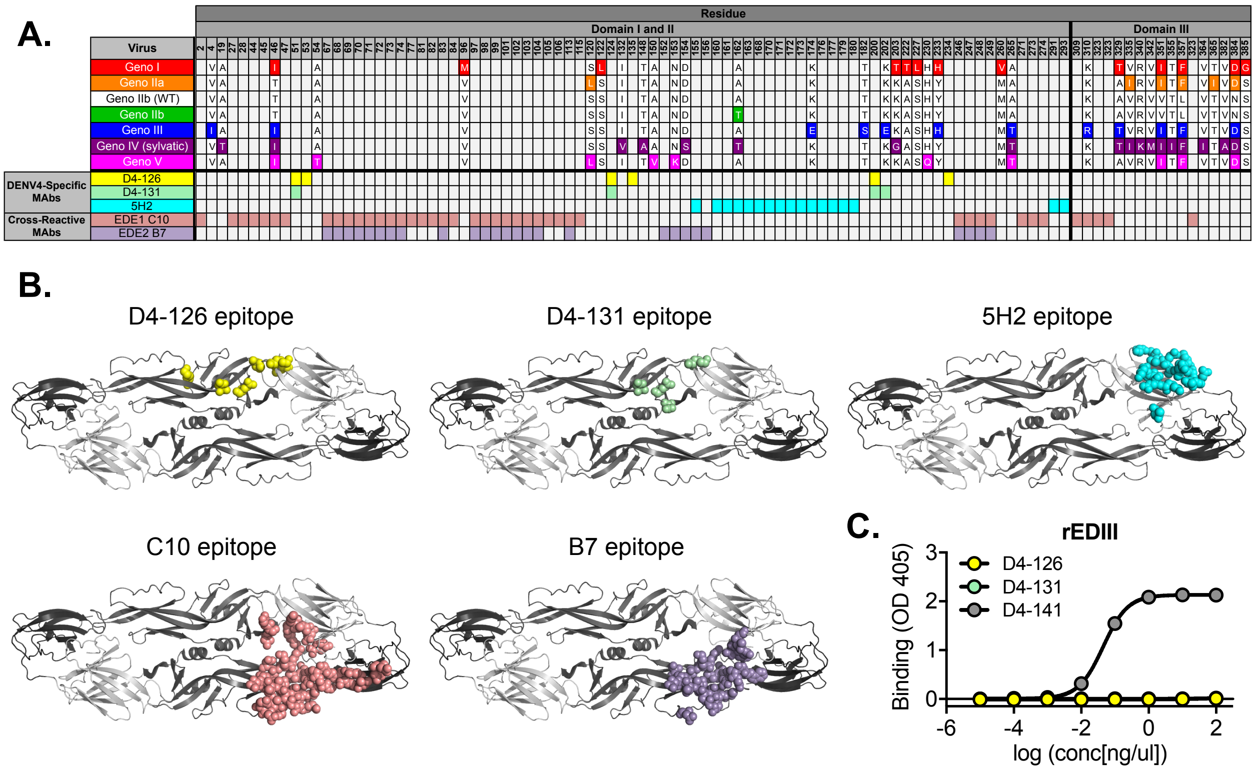

Supplemental Figure 4. Epitopes of DENV serotype-specific and cross-reactive antibodies. Related to Figures 5-6. A) Amino acid sequences of DENV4 genotype viruses with known monoclonal antibodies contact residues highlighted. B) Monoclonal antibody contact residues are mapped onto the envelope dimer (PBD = 1OAN). C) Monoclonal antibody D4-141 binds recombinant envelope domain III (rEDIII) protein.

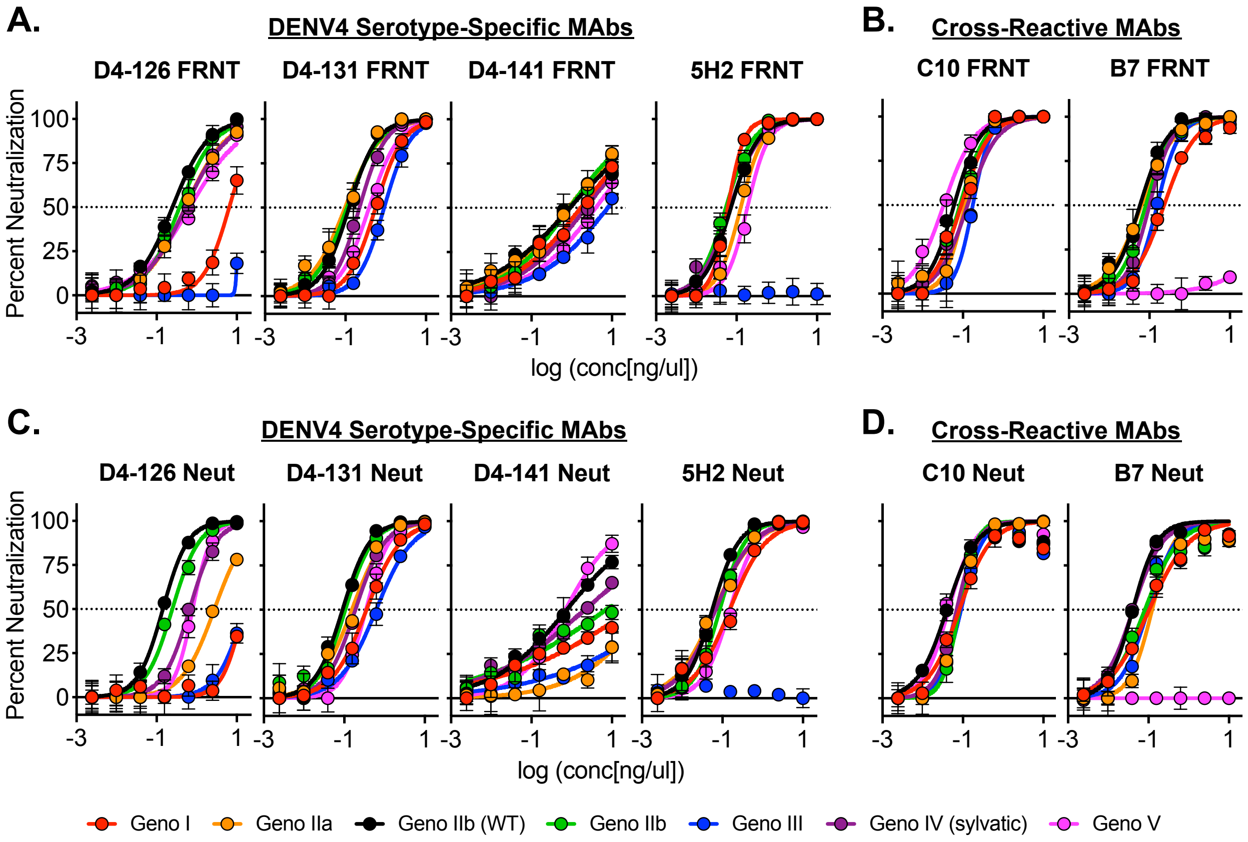

Supplemental Figure 5. Monoclonal antibody neutralization curves. Related to Figure 6. DENV4 serotype-specific antibodies D4-126, D4-131, D4-141 and 5H2 and DENV cross-reactive antibodies C10 and B7 were evaluated for their ability to neutralize DENV4 genotype viruses in a A) Vero cell focus reduction neutralization test (FRNT) and B) flow-cytometery-based neutralization assay (Neut) (mean ± SD of biological triplicates).

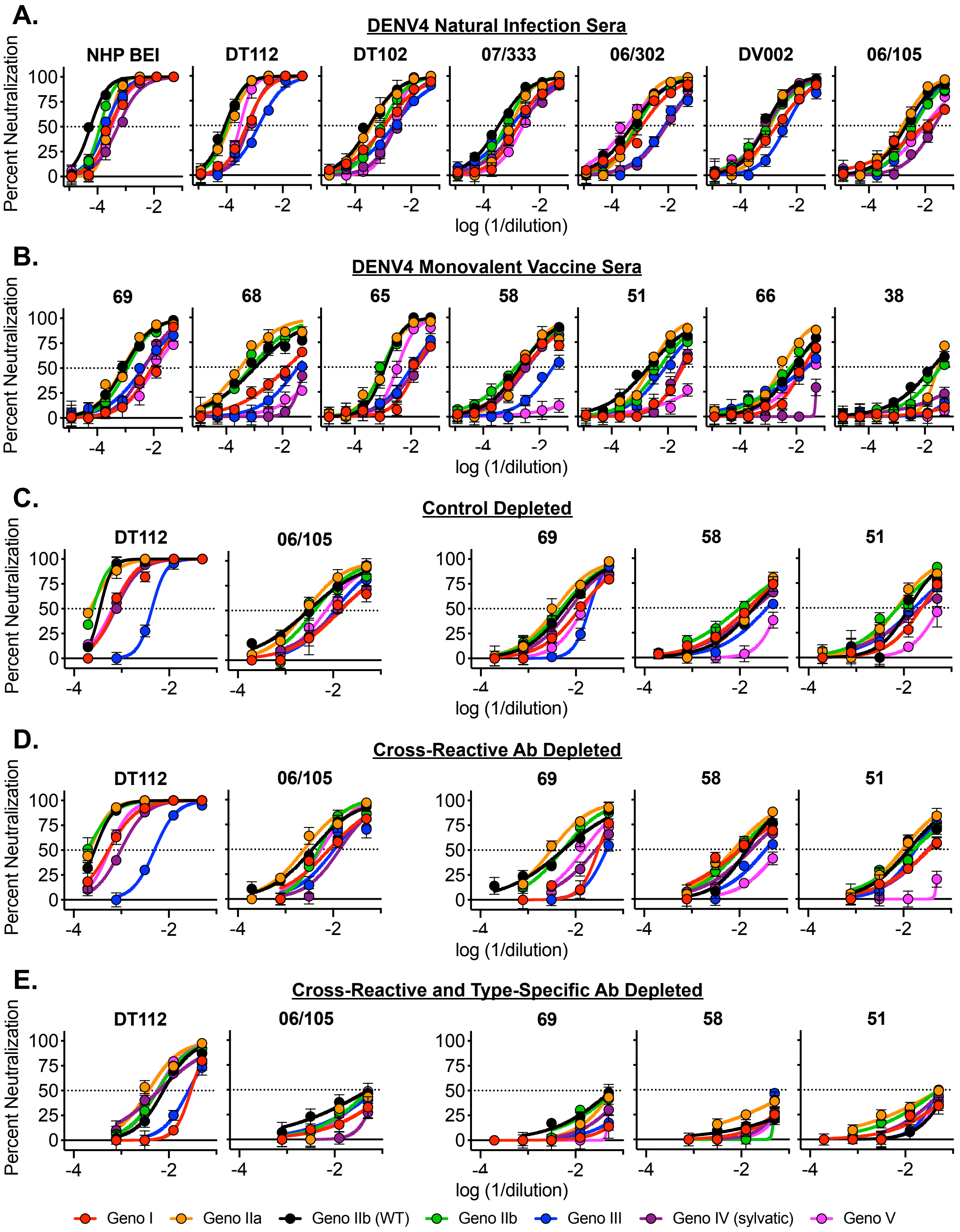

Supplemental Figure 6. DENV4 natural infection and monovalent vaccine immune sera neutralization curves. Related to Figure 7. Using Vero cell focus reduction neutralization test (FRNT), A) pooled polyclonal immune sera

from DENV4 infected non-human primates (NHP) or from naturally infected individuals, or from B) individuals who received the NIH DENV4 monovalent vaccine were evaluated for their ability to neutralize DENV4 genotype viruses. DENV4 natural infection and monovalent vaccine sera were C) control depleted with BSA, D) depleted of cross-reactive antibodies, or E) depleted of all DENV antibodies and evaluated for their ability to neutralize DENV4 genotype viruses (mean ± SD of biological duplicates).

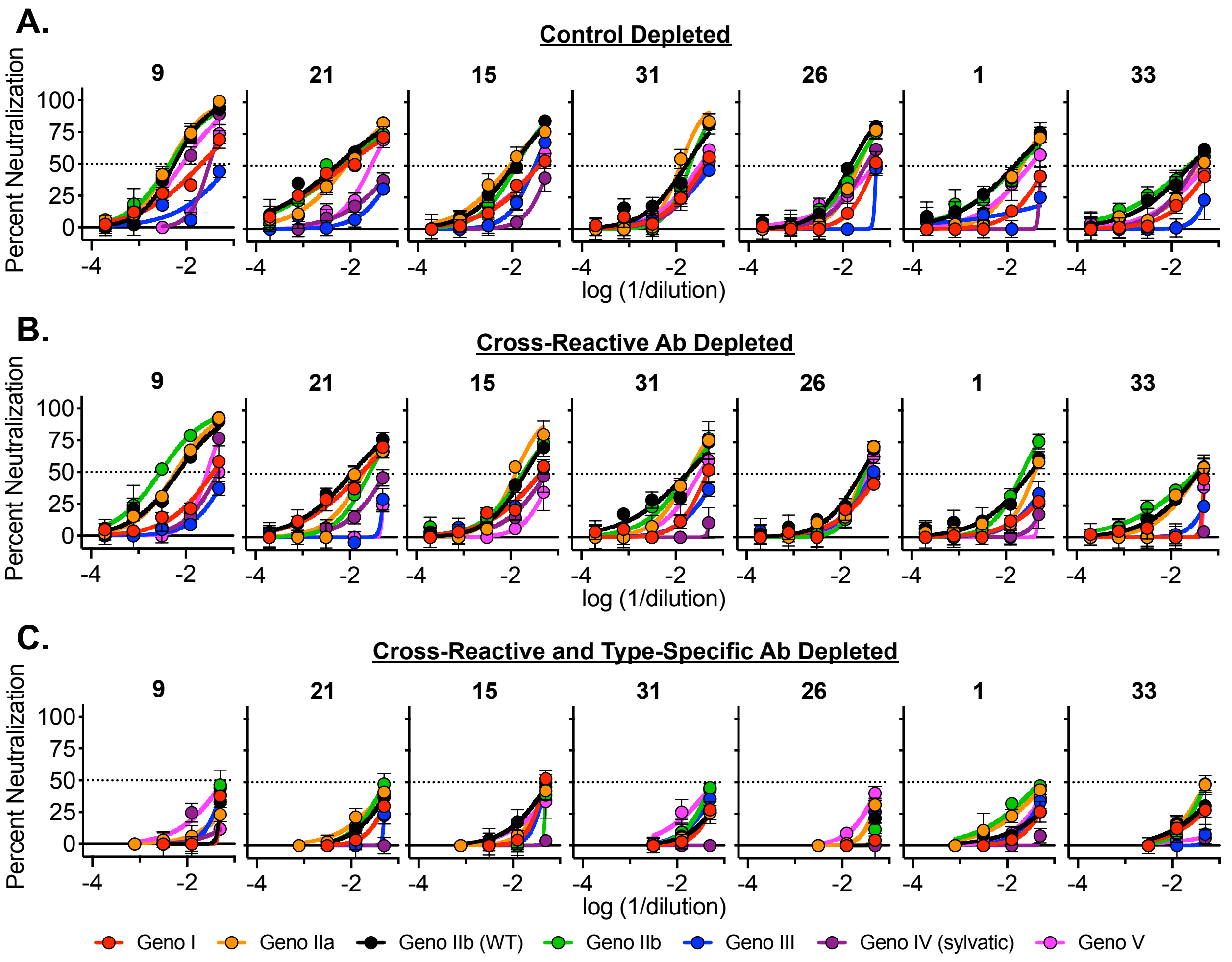

Supplemental Figure 7. DENV tetravalent vaccine immune sera neutralization curves. Related to Figure 7. Using Vero cell focus reduction neutralization test (FRNT), DENV tetravalent immune sera were A) control depleted with BSA, B) depleted of cross-reactive antibodies, or C) depleted of all DENV antibodies and evaluated for their ability to neutralize DENV4 genotype viruses (mean ± SD of biological duplicates).

Genotype	Location	Year	Accession Number
Genotype I	Cambodia	2010	KF543272.1
Genotype IIa	French Polynesia	2009	JN832541.1
Genotype IIb	Puerto Rico	1999	FJ882599.1
Genotype IIb (WT)	Sri Lanka	1992	KJ160504.1
Genotype III	Thailand	2001	AY618940.1
Genotype IV	Malaysia	1973	AF231724.1
Genotype V	India	1961	JF262783.1

Supplemental Table 1. Viruses in recombinant DENV4 variant panel. Related to Figures 1-2.

Name	Description	Region	Genotype
D4/13DX3-741_JX644006	Dengue virus 4 strain 13DX3-741 envelope protein gene, partial cds	Vietnam	I
D4/13DX3-773_JX644009	Dengue virus 4 strain 13DX3-773 envelope protein gene, partial cds	Vietnam	I
D4/13DX3-779_JX644008	Dengue virus 4 strain 13DX3-779 envelope protein gene, partial cds	Vietnam	I
D4/13DX4-413_JX644007	Dengue virus 4 strain 13DX4-413 envelope protein gene, partial cds	Vietnam	I
D4/13DX4-417_JX644010	Dengue virus 4 strain 13DX4-417 envelope protein gene, partial cds	Vietnam	I
D4/13DX4-446_JX644011	Dengue virus 4 strain 13DX4-446 envelope protein gene, partial cds	Vietnam	I
D4/13DX4-452_JX644012	Dengue virus 4 strain 13DX4-452 envelope protein gene, partial cds	Vietnam	I
D4/China/YN/15DGR284/2015_KX262925	Dengue virus 4 isolate DENV-4/China/YN/15DGR284 (2015) envelope protein gene, partial cds	China	I
D4/China/YN/15DGR32/2015_KX262923	Dengue virus 4 isolate DENV-4/China/YN/15DGR32 (2015) envelope protein gene, partial cds	China	I
D4/China/YN/15DGR34/2015_KX262920	Dengue virus 4 isolate DENV-4/China/YN/15DGR34 (2015) envelope protein gene, partial cds	China	I
D4/China/YN/15DGR35/2015_KX262921	Dengue virus 4 isolate DENV-4/China/YN/15DGR35 (2015) envelope protein gene, partial cds	China	I
D4/China/YN/15DGR394/2015_KX262926	Dengue virus 4 isolate DENV-4/China/YN/15DGR394 (2015) envelope protein gene, partial cds	China	I
D4/China/YN/15DGR50/2015_KX262924	Dengue virus 4 isolate DENV-4/China/YN/15DGR50 (2015) envelope protein gene, partial cds	China	I
D4/China/YN/15DGR9/2015_KX262922	Dengue virus 4 isolate DENV-4/China/YN/15DGR9 (2015) envelope protein gene, partial cds	China	I
D4/H781363_JQ513345	Dengue virus 4 strain H781363, complete genome	Brazil	I
D4/Hu/Cambodia/NRTqs/2013_AB873105	Dengue virus 4 gene for envelope protein, partial cds, strain: D4/Hu/Cambodia/NRTqs/2013	Cambodia	I
D4/SG(EHI)D4/02990Y14_KX224312	Dengue virus 4 isolate SG(EHI)D4/02990Y14, complete genome	Singapore	I
D4/U0811386_KF543272	Dengue virus 4 isolate U0811386 polyprotein gene, partial cds	Cambodia	I
D4/CO/BID-V3406/2001_GQ868579	Dengue virus 4 isolate DENV-4/CO/BID-V3406/2001, complete genome	Colombia	IIa
D4/CO/BID-V3410/2004_GQ868583	Dengue virus 4 isolate DENV-4/CO/BID-V3410/2004, complete genome	Colombia	IIa
D4/CO/BID-V3411/2004_GQ868584	Dengue virus 4 isolate DENV-4/CO/BID-V3411/2004, complete genome	Colombia	IIa
D4/CO/BID-V3412/2005_GQ868585	Dengue virus 4 isolate DENV-4/CO/BID-V3412/2005, complete genome	Colombia	IIa
D4/H772846_JQ513330	Dengue virus 4 strain H772846, complete genome	Brazil	IIa
D4/H772852_JQ513331	Dengue virus 4 strain H772852, complete genome	Brazil	IIa
D4/H772854_JN559741	Dengue virus 4 strain H772854, complete genome	Brazil	IIa
D4/H773583_JQ513332	Dengue virus 4 strain H773583, complete genome	Brazil	IIa
D4/H774846_JQ513333	Dengue virus 4 strain H774846, complete genome	Brazil	IIa
D4/H779228_JQ513338	Dengue virus 4 strain H779228, complete genome	Brazil	IIa
D4/H779652_JQ513339	Dengue virus 4 strain H779652, complete genome	Brazil	IIa
D4/H780090_JQ513340	Dengue virus 4 strain H780090, complete genome	Brazil	IIa
D4/H780120_JQ513341	Dengue virus 4 strain H780120, complete genome	Brazil	IIa
D4/H780556_JQ513342	Dengue virus 4 strain H780556, complete genome	Brazil	IIa
D4/H780563_JQ513343	Dengue virus 4 strain H780563, complete genome	Brazil	IIa
D4/H780571_JQ513344	Dengue virus 4 strain H780571, complete genome	Brazil	IIa
D4/MT/BR12_TVP17898/2012_KJ579243	Dengue virus 4 strain DENV-4/MT/BR12_TVP17898/2012 isolate serum_12, complete genome	Brazil	IIa
D4/MT/BR2_TVP17888/2012_KJ579240	Dengue virus 4 strain DENV-4/MT/BR2_TVP17888/2012 isolate serum_2, complete genome	Brazil	IIa
D4/MT/BR20_TVP17906/2012_KJ579244	Dengue virus 4 strain DENV-4/MT/BR20_TVP17906/2012 isolate serum_20, complete genome	Brazil	IIa
D4/MT/BR23_TVP17909/2012_KJ579245	Dengue virus 4 strain DENV-4/MT/BR23_TVP17909/2012 isolate serum_23, complete genome	Brazil	IIa
D4/MT/BR24_TVP17910/2012_KJ579246	Dengue virus 4 strain DENV-4/MT/BR24_TVP17910/2012 isolate serum_24, complete genome	Brazil	IIa
D4/MT/BR27_TVP17913/2012_KJ579247	Dengue virus 4 strain DENV-4/MT/BR27_TVP17913/2012 isolate serum_27, complete genome	Brazil	IIa
D4/MT/BR28_TVP17914/2012_KJ579248	Dengue virus 4 strain DENV-4/MT/BR28_TVP17914/2012 isolate serum_28, complete genome	Brazil	IIa
D4/MT/BR33_TVP17919/2012_KJ596658	Dengue virus 4 strain DENV-4/MT/BR33_TVP17919/2012 isolate serum_33, complete genome	Brazil	IIa
D4/MT/BR35_TVP17921/2012_KJ596659	Dengue virus 4 strain DENV-4/MT/BR35_TVP17921/2012 isolate serum_35, complete genome	Brazil	IIa
D4/MT/BR40_TVP17926/2012_KJ596660	Dengue virus 4 strain DENV-4/MT/BR40_TVP17926/2012 isolate serum_40, complete genome	Brazil	IIa
D4/MT/BR44_TVP17930/2012_KJ596661	Dengue virus 4 strain DENV-4/MT/BR44_TVP17930/2012 isolate serum_44, complete genome	Brazil	IIa
D4/MT/BR47_TVP17933/2012_KJ596662	Dengue virus 4 strain DENV-4/MT/BR47_TVP17933/2012 isolate serum_47, complete genome	Brazil	IIa
D4/MT/BR48_TVP17934/2012_KJ596663	Dengue virus 4 strain DENV-4/MT/BR48_TVP17934/2012 isolate serum_48, complete genome	Brazil	IIa
D4/MT/BR50_TVP18148/2012_KJ596664	Dengue virus 4 strain DENV-4/MT/BR50_TVP18148/2012 isolate serum_50, complete genome	Brazil	IIa
D4/MT/BR52_TVP17938/2012_KJ596665	Dengue virus 4 strain DENV-4/MT/BR52_TVP17938/2012 isolate serum_52, complete genome	Brazil	IIa
D4/MT/BR53_TVP17939/2012_KJ596666	Dengue virus 4 strain DENV-4/MT/BR53_TVP17939/2012 isolate serum_53, complete genome	Brazil	IIa
D4/MT/BR55_TVP17941/2012_KJ596667	Dengue virus 4 strain DENV-4/MT/BR55_TVP17941/2012 isolate serum_55, complete genome	Brazil	IIa
D4/MT/BR60_TVP17946/2012_KJ596668	Dengue virus 4 strain DENV-4/MT/BR60_TVP17946/2012 isolate serum_60, complete genome	Brazil	IIa
D4/MT/BR73_TVP17951/2012_KJ596669	Dengue virus 4 strain DENV-4/MT/BR73_TVP17951/2012 isolate serum_73, complete genome	Brazil	IIa
D4/MT/BR76_TVP17953/2012_KJ596670	Dengue virus 4 strain DENV-4/MT/BR76_TVP17953/2012 isolate serum_76, complete genome	Brazil	IIa
D4/MT/BR8_TVP17894/2012_KJ579241	Dengue virus 4 strain DENV-4/MT/BR8_TVP17894/2012 isolate serum_8, complete genome	Brazil	IIa
D4/MT/BR84_TVP17961/2012_KJ596671	Dengue virus 4 strain DENV-4/MT/BR84_TVP17961/2012 isolate serum_84, complete genome	Brazil	IIa
D4/MT/BR9_TVP17895/2012_KJ579242	Dengue virus 4 strain DENV-4/MT/BR9_TVP17895/2012 isolate serum_9, complete genome	Brazil	IIa
D4/MT/BR91_TVP17968/2012_KJ596672	Dengue virus 4 strain DENV-4/MT/BR91_TVP17968/2012 isolate serum_91, complete genome	Brazil	IIa
D4/MT/BR92_TVP17969/2012_KJ596673	Dengue virus 4 strain DENV-4/MT/BR92_TVP17969/2012 isolate serum_92, complete genome	Brazil	IIa
D4/MT/BR94_TVP17971/2012_KJ596674	Dengue virus 4 strain DENV-4/MT/BR94_TVP17971/2012 isolate serum_94, complete genome	Brazil	IIa
D4/PF09/220709-54_JN832541	Dengue virus 4 isolate PF09/220709-54 envelope protein (E) gene, partial cds	French Polynesia	IIa
D4/SG(EHI)D4/20293Y13_KX224301	Dengue virus 4 isolate SG(EHI)D4/20293Y13 envelope protein gene, partial cds	Singapore	IIa
D4/SG(EHI)D4/21661Y13_KX224302	Dengue virus 4 isolate SG(EHI)D4/21661Y13 envelope protein gene, partial cds	Singapore	IIa
D4/SG(EHI)D4/25974Y13_KX224304	Dengue virus 4 isolate SG(EHI)D4/25974Y13 envelope protein gene, partial cds	Singapore	IIa
D4/SG(EHI)D4/30313Y13_KX224311	Dengue virus 4 isolate SG(EHI)D4/30313Y13 envelope protein gene, partial cds	Singapore	IIa
D4/SG/06K2270DK1/2005_GQ398256	Dengue virus 4 strain DENV-4/SG/06K2270DK1/2005, complete genome	Singapore	IIa
D4/VE/BID-V1153/2007_GQ868642	Dengue virus 4 isolate DENV-4/VE/BID-V1153/2007, complete genome	Venezuela	IIa
D4/VE/BID-V1154/2007_GQ868643	Dengue virus 4 isolate DENV-4/VE/BID-V1154/2007, complete genome	Venezuela	IIa
D4/VE/BID-V1155/2007_GQ868644	Dengue virus 4 isolate DENV-4/VE/BID-V1155/2007, complete genome	Venezuela	IIa
D4/VE/BID-V1156/2007_GQ868645	Dengue virus 4 isolate DENV-4/VE/BID-V1156/2007, complete genome	Venezuela	IIa
D4/VE/BID-V1157/2007_EU854299	Dengue virus 4 isolate DENV-4/VE/BID-V1157/2007, complete genome	Venezuela	IIa
D4/VE/BID-V1158/2007_FJ182016	Dengue virus 4 isolate DENV-4/VE/BID-V1158/2007, complete genome	Venezuela	IIa
D4/VE/BID-V1159/2007_EU854300	Dengue virus 4 isolate DENV-4/VE/BID-V1159/2007, complete genome	Venezuela	IIa
D4/VE/BID-V1160/2007_FJ182017	Dengue virus 4 isolate DENV-4/VE/BID-V1160/2007, complete genome	Venezuela	IIa
D4/VE/BID-V1161/2007_EU854301	Dengue virus 4 isolate DENV-4/VE/BID-V1161/2007, complete genome	Venezuela	IIa
D4/VE/BID-V2163/1998_FJ639736	Dengue virus 4 isolate DENV-4/VE/BID-V2163/1998, complete genome	Venezuela	IIa
D4/VE/BID-V2164/1998_FJ639737	Dengue virus 4 isolate DENV-4/VE/BID-V2164/1998, complete genome	Venezuela	IIa
D4/VE/BID-V2165/1998_FJ639738	Dengue virus 4 isolate DENV-4/VE/BID-V2165/1998, complete genome	Venezuela	IIa
D4/VE/BID-V2166/1998_FJ639739	Dengue virus 4 isolate DENV-4/VE/BID-V2166/1998, complete genome	Venezuela	IIa
D4/VE/BID-V2167/1998_JN819409	Dengue virus 4 isolate DENV-4/VE/BID-V2167/1998, complete genome	Venezuela	IIa
D4/VE/BID-V2170/1999_FJ639742	Dengue virus 4 isolate DENV-4/VE/BID-V2170/1999, complete genome	Venezuela	IIa
D4/VE/BID-V2172/1999_FJ639744	Dengue virus 4 isolate DENV-4/VE/BID-V2172/1999, complete genome	Venezuela	IIa
D4/VE/BID-V2173/1999_FJ639745	Dengue virus 4 isolate DENV-4/VE/BID-V2173/1999, complete genome	Venezuela	IIa
D4/VE/BID-V2176/2000_FJ850095	Dengue virus 4 isolate DENV-4/VE/BID-V2176/2000, complete genome	Venezuela	IIa
D4/VE/BID-V2177/2000_FJ639748	Dengue virus 4 isolate DENV-4/VE/BID-V2177/2000, complete genome	Venezuela	IIa
D4/VE/BID-V2194/2001_FJ639764	Dengue virus 4 isolate DENV-4/VE/BID-V2194/2001, complete genome	Venezuela	IIa
D4/VE/BID-V2206/2001_FJ639773	Dengue virus 4 isolate DENV-4/VE/BID-V2206/2001, complete genome	Venezuela	IIa
D4/VE/BID-V2489/2007_FJ882580	Dengue virus 4 isolate DENV-4/VE/BID-V2489/2007, complete genome	Venezuela	IIa

D4/VE/BID-V2490/2007_FJ882581	Dengue virus 4 isolate DENV-4/VE/BID-V2490/2007, complete genome	Venezuela	IIa
D4/VE/BID-V2491/2007_FJ882582	Dengue virus 4 isolate DENV-4/VE/BID-V2491/2007, complete genome	Venezuela	IIa
D4/VE/BID-V2492/2007_FJ882583	Dengue virus 4 isolate DENV-4/VE/BID-V2492/2007, complete genome	Venezuela	IIa
D4/VE/BID-V2493/200_FJ882584	Dengue virus 4 isolate DENV-4/VE/BID-V2493/2007, complete genome	Venezuela	IIa
D4/VE/BID-V2494/2007_FJ882585	Dengue virus 4 isolate DENV-4/VE/BID-V2494/2007, complete genome	Venezuela	IIa
D4/VE/BID-V2495/2007_FJ882586	Dengue virus 4 isolate DENV-4/VE/BID-V2495/2007, complete genome	Venezuela	IIa
D4/VE/BID-V2496/2007_FJ882587	Dengue virus 4 isolate DENV-4/VE/BID-V2496/2007, complete genome	Venezuela	IIa
D4/VE/BID-V2497/2007_FJ882588	Dengue virus 4 isolate DENV-4/VE/BID-V2497/2007, complete genome	Venezuela	IIa
D4/VE/BID-V2498/2007_FJ882589	Dengue virus 4 isolate DENV-4/VE/BID-V2498/2007, complete genome	Venezuela	IIa
D4/VE/BID-V2499/2007_FJ882590	Dengue virus 4 isolate DENV-4/VE/BID-V2499/2007, complete genome	Venezuela	IIa
D4/VE/BID-V2500/2007_FJ882591	Dengue virus 4 isolate DENV-4/VE/BID-V2500/2007, complete genome	Venezuela	IIa
D4/VE/BID-V2501/2008_FJ882592	Dengue virus 4 isolate DENV-4/VE/BID-V2501/2008, complete genome	Venezuela	IIa
D4/VE/BID-V2607/2006_JN819406	Dengue virus 4 isolate DENV-4/VE/BID-V2607/2006, complete genome	Venezuela	IIa
D4/VE/BID-V2610/2007_GQ199876	Dengue virus 4 isolate DENV-4/VE/BID-V2610/2007, complete genome	Venezuela	IIa
D4/ARC-1-13_KF809760	Dengue virus 4 strain ARC-1-13 envelope protein gene, partial cds	Puerto Rico	IIb
D4/ARC-33-13_KF809761	Dengue virus 4 strain ARC-33-13 envelope protein gene, partial cds	Puerto Rico	IIb
D4/ARC-55-12_KF809754	Dengue virus 4 strain ARC-55-12 envelope protein gene, partial cds	Puerto Rico	IIb
D4/ARC-65-12_KF809755	Dengue virus 4 strain ARC-65-12 envelope protein gene, partial cds	Puerto Rico	IIb
D4/ARC-65-13_KF809762	Dengue virus 4 strain ARC-65-13 envelope protein gene, partial cds	Puerto Rico	IIb
D4/ARC-69-12_KF809756	Dengue virus 4 strain ARC-69-12 envelope protein gene, partial cds	Puerto Rico	IIb
D4/ARC-75-12_KF809757	Dengue virus 4 strain ARC-75-12 envelope protein gene, partial cds	Puerto Rico	IIb
D4/ARC-78-12_KF809758	Dengue virus 4 strain ARC-78-12 envelope protein gene, partial cds	Puerto Rico	IIb
D4/ARC-80-12_KF809759	Dengue virus 4 strain ARC-80-12 envelope protein gene, partial cds	Puerto Rico	IIb
D4/CO/BID-V1600/1997_FJ024476	Dengue virus 4 isolate DENV-4/CO/BID-V1600/1997, complete genome	Colombia	IIb
D4/CO/BID-V3407/2001_GQ868580	Dengue virus 4 isolate DENV-4/CO/BID-V3407/2001, complete genome	Colombia	IIb
D4/CO/BID-V3408/2001_GQ868581	Dengue virus 4 isolate DENV-4/CO/BID-V3408/2001, complete genome	Colombia	IIb
D4/CO/BID-V3409/2001_GQ868582	Dengue virus 4 isolate DENV-4/CO/BID-V3409/2001, complete genome	Colombia	IIb
D4/H402276_JN559740	Dengue virus 4 strain H402276, complete genome	Brazil	IIb
D4/H775222_JQ513334	Dengue virus 4 strain H775222, complete genome	Brazil	IIb
D4/H778494_JQ513335	Dengue virus 4 strain H778494, complete genome	Brazil	IIb
D4/H778504_JQ513336	Dengue virus 4 strain H778504, complete genome	Brazil	IIb
D4/H778887_JQ513337	Dengue virus 4 strain H778887, complete genome	Brazil	IIb
D4/rDENV4_KJ160504	Dengue virus 4 isolate rDENV4, complete genome	Sri Lanka	IIb
D4/US/BID-V1082/1998_FJ024424	Dengue virus 4 isolate DENV-4/US/BID-V1082/1998, complete genome	USA	IIb
D4/US/BID-V1083/1986_EU854295	Dengue virus 4 isolate DENV-4/US/BID-V1083/1986, complete genome	USA	IIb
D4/US/BID-V1093/1998_EU854296	Dengue virus 4 isolate DENV-4/US/BID-V1093/1998, complete genome	USA	IIb
D4/US/BID-V1094/1998_EU854297	Dengue virus 4 isolate DENV-4/US/BID-V1094/1998, complete genome	USA	IIb
D4/US/BID-V2429/1994_GQ199878	Dengue virus 4 isolate DENV-4/US/BID-V2429/1994, complete genome	USA	IIb
D4/US/BID-V2430/1994_GQ199879	Dengue virus 4 isolate DENV-4/US/BID-V2430/1994, complete genome	USA	IIb
D4/US/BID-V2431/1995_GQ199880	Dengue virus 4 isolate DENV-4/US/BID-V2431/1995, complete genome	USA	IIb
D4/US/BID-V2432/1995_GQ252675	Dengue virus 4 isolate DENV-4/US/BID-V2432/1995, complete genome	USA	IIb
D4/US/BID-V2433/1995_FJ810417	Dengue virus 4 isolate DENV-4/US/BID-V2433/1995, complete genome	USA	IIb
D4/US/BID-V2434/1995_FJ850057	Dengue virus 4 isolate DENV-4/US/BID-V2434/1995, complete genome	USA	IIb
D4/US/BID-V2435/1996_GQ199881	Dengue virus 4 isolate DENV-4/US/BID-V2435/1996, complete genome	USA	IIb
D4/US/BID-V2436/1996_GQ199882	Dengue virus 4 isolate DENV-4/US/BID-V2436/1996, complete genome	USA	IIb
D4/US/BID-V2437/1996_GQ199883	Dengue virus 4 isolate DENV-4/US/BID-V2437/1996, complete genome	USA	IIb
D4/US/BID-V2438/1996_GQ199884	Dengue virus 4 isolate DENV-4/US/BID-V2438/1996, complete genome	USA	IIb
D4/US/BID-V2439/1996_GQ199885	Dengue virus 4 isolate DENV-4/US/BID-V2439/1996, complete genome	USA	IIb
D4/US/BID-V2440/1996_FJ850058	Dengue virus 4 isolate DENV-4/US/BID-V2440/1996, complete genome	USA	IIb
D4/US/BID-V2441/1998_FJ882595	Dengue virus 4 isolate DENV-4/US/BID-V2441/1998, complete genome	USA	IIb
D4/US/BID-V2442/1998_FJ882596	Dengue virus 4 isolate DENV-4/US/BID-V2442/1998, complete genome	USA	IIb
D4/US/BID-V2443/1998_FJ850059	Dengue virus 4 isolate DENV-4/US/BID-V2443/1998, complete genome	USA	IIb
D4/US/BID-V2444/1998_FJ882597	Dengue virus 4 isolate DENV-4/US/BID-V2444/1998, complete genome	USA	IIb
D4/US/BID-V2445/1998_FJ882598	Dengue virus 4 isolate DENV-4/US/BID-V2445/1998, complete genome	USA	IIb
D4/US/BID-V2446/1999_FJ882599	Dengue virus 4 isolate DENV-4/US/BID-V2446/1999, complete genome	USA	IIb
D4/US/BID-V2447/1999_FJ882600	Dengue virus 4 isolate DENV-4/US/BID-V2447/1999, complete genome	USA	IIb
D4/US/BID-V2448/1999_FJ882601	Dengue virus 4 isolate DENV-4/US/BID-V2448/1999, complete genome	USA	IIb
D4/US/BID-V860/1994_FJ226067	Dengue virus 4 isolate DENV-4/US/BID-V860/1994, complete genome	USA	IIb
D4/0017/1997_AY618978	Dengue virus type 4 strain ThD4_0017_97 envelope protein (E) gene, partial cds	Thailand	III
D4/0164/1999_AY618986	Dengue virus type 4 strain ThD4_0164_99 envelope protein (E) gene, partial cds	Thailand	III
D4/0439/2001_AY618940	Dengue virus type 4 strain ThD4_0439_01 envelope protein (E) gene, partial cds	Thailand	III
D4/0476/1997_AY618979	Dengue virus type 4 strain ThD4_0476_97 envelope protein (E) gene, partial cds	Thailand	III
D4/1270/1998_AY618981	Dengue virus type 4 strain ThD4_1270_98 envelope protein (E) gene, partial cds	Thailand	III
D4/P73-1120_AF231724	Dengue virus type 4 isolate P73-1120 envelope protein (E) gene, partial cds	Malaysia	IV (sylvatic)
D4/P75-215_AF231725	Dengue virus type 4 isolate P75-215 envelope protein (E) gene, partial cds	Malaysia	IV (sylvatic)
D4/P75-514_AF231723	Dengue virus type 4 isolate P75-514 envelope protein (E) gene, partial cds	Malaysia	IV (sylvatic)
D4/Hu/Thailand/1963_DVU18440	Dengue virus type 4 Thailand 1963 polyprotein gene, partial cds	Thailand	V
D4/IND/0952326/2009_JQ922560	Dengue virus 4 isolate DENV-4/IND/0952326/2009, complete genome	India	V
D4/IND/624000/1962_JQ922558	Dengue virus 4 isolate DENV-4/IND/624000/1962, complete genome	India	V
D4/IND/793679/1979_JQ922559	Dengue virus 4 isolate DENV-4/IND/793679/1979, complete genome	India	V
D4/INDIA-G11337_JF262783	Dengue virus 4 isolate INDIA G11337, complete genome	India	V
D4/KBPV-VR-31_KP406806	Dengue virus 4 isolate DENV-4/KBPV-VR-31, complete genome	South Korea	V
D4/NIV-611319_JQ686059	Dengue virus 4 strain NIV_611319 envelope protein gene, partial cds	India	V
D4/NIV-62231_JQ686066	Dengue virus 4 strain NIV_62231 envelope protein gene, partial cds	India	V
D4/NIV-62235_JQ686065	Dengue virus 4 strain NIV_62235 envelope protein gene, partial cds	India	V
D4/NIV-624000_JQ686064	Dengue virus 4 strain NIV_624000 envelope protein gene, partial cds	India	V
D4/NIV-631302_JQ686057	Dengue virus 4 strain NIV_631302 envelope protein gene, partial cds	India	V
D4/NIV-64431_JQ686060	Dengue virus 4 strain NIV_64431 envelope protein gene, partial cds	India	V
D4/NIV-654021_JQ686062	Dengue virus 4 strain NIV_654021 envelope protein gene, partial cds	India	V
D4/NIV-654129-2_JQ686061	Dengue virus 4 strain NIV_654129-2 envelope protein gene, partial cds	India	V
D4/NIV-793679_JQ686058	Dengue virus 4 strain NIV_793679 envelope protein gene, partial cds	India	V
D4/PH/BID-V3361/1956_GQ868594	Dengue virus 4 isolate DENV-4/PH/BID-V3361/1956, complete genome	Philippines	V

Supplemental Table 2. DENV4 virus sequences in phylogenetic analyses. Related to Figure 1. All sequences used to generate phylogenetic tree in Figure 1 are listed by sequence name, description, location of collection and genotype.

**Name**	**Asn-67 Gly Site**	**Asn-153 Gly Site**	**Asn-153 Seq.**
D4/Hu/Thailand/1963_DVU18440	yes	**no**	**NDI**
D4/IND/0952326/2009_JQ922560	yes	yes	NDT
D4/IND/624000/1962_JQ922558	yes	**no**	**NDI**
D4/IND/793679/1979_JQ922559	yes	**no**	**NDI**
D4/INDIA-G11337_JF262783	yes	**no**	**KDT**
D4/KBPV-VR-31_KP406806	yes	**no**	**NDI**
D4/NIV-611319_JQ686059	yes	**no**	**NDI**
D4/NIV-62231_JQ686066	yes	**no**	**NDI**
D4/NIV-62235_JQ686065	yes	**no**	**KDT**
D4/NIV-624000_JQ686064	yes	**no**	**NDI**
D4/NIV-631302_JQ686057	yes	**no**	**NDI**
D4/NIV-64431_JQ686060	yes	**no**	**KDT**
D4/NIV-654021_JQ686062	yes	yes	NDT
D4/NIV-654129-2_JQ686061	yes	**no**	**NDI**
D4/NIV-793679_JQ686058	yes	**no**	**NDI**
D4/PH/BID-V3361/1956_GQ868594	yes	**no**	**NDI**
		**87.5% missing gly site**	

Supplemental Table 3. Glycosylation site sequences of all genotype V sequences. Related to Figure 4. 16 genotype V sequences used to generate phylogenetic tree in Figure 1 were evaluated to determine if glycosylation sites were present at positions 67 and 153. Fourteen of 16 sequences are missing glycosylation motif (N-X-T/S) at position 153.
